# Efficacy of Urtoxazumab (TMA-15 Humanized Monoclonal Antibody Specific for Shiga Toxin 2) Against Post-Diarrheal Neurological Sequelae Caused by *Escherichia coli* O157:H7 Infection in the Neonatal Gnotobiotic Piglet Model

**DOI:** 10.3390/toxins9020049

**Published:** 2017-01-26

**Authors:** Rodney A. Moxley, David H. Francis, Mizuho Tamura, David B. Marx, Kristina Santiago-Mateo, Mojun Zhao

**Affiliations:** 1 School of Veterinary Medicine and Biomedical Sciences, University of Nebraska-Lincoln, Lincoln, NE 68583, USA; 2 Department of Veterinary and Biomedical Sciences, South Dakota State University, Brookings, SD 57007, USA; David.Francis@sdstate.edu; 3 Teijin Pharma Limited, Pharmacology Research Department, 4-3-2 Asahigaoka, Hino, Tokyo 191-8512, Japan; miz.tamura@teijin.co.jp; 4 Department of Statistics, University of Nebraska-Lincoln, Lincoln, NE 68583, USA; dmarx1@unl.edu; 5 Canadian Food Inspection Agency, Lethbridge Laboratory, Box 640 TWP Rd 9-1, Lethbridge, AB T1J 3Z4, Canada; Kristina.Santiago-Mateo@inspection.gc.ca; 6 Valley Pathologists, Inc., 1100 South Main Street, Suite 308, Dayton, OH 45409, USA; zhaomojun@gmail.com

**Keywords:** Shiga toxin, enterohemorrhagic *E. coli*, gnotobiotic piglets, monoclonal antibody

## Abstract

Enterohemorrhagic *Escherichia coli* (EHEC) is the most common cause of hemorrhagic colitis and hemolytic uremic syndrome in human patients, with brain damage and dysfunction the main cause of acute death. We evaluated the efficacy of urtoxazumab (TMA-15, Teijin Pharma Limited), a humanized monoclonal antibody against Shiga toxin (Stx) 2 for the prevention of brain damage, dysfunction, and death in a piglet EHEC infection model. Forty-five neonatal gnotobiotic piglets were inoculated orally with 3 × 10^9^ colony-forming units of EHEC O157:H7 strain EDL933 (Stx1^+^, Stx2^+^) when 22–24 h old. At 24 h post-inoculation, piglets were intraperitoneally administered placebo or TMA-15 (0.3, 1.0 or 3.0 mg/kg body weight). Compared to placebo (*n* = 10), TMA-15 (*n* = 35) yielded a significantly greater probability of survival, length of survival, and weight gain (*p* <0.05). The efficacy of TMA-15 against brain lesions and death was 62.9% (*p* = 0.0004) and 71.4% (*p* = 0.0004), respectively. These results suggest that TMA-15 may potentially prevent or reduce vascular necrosis and infarction of the brain attributable to Stx2 in human patients acutely infected with EHEC. However, we do not infer that TMA-15 treatment will completely protect human patients infected with EHEC O157:H7 strains that produce both Stx1 and Stx2.

## 1. Introduction

Strains of Shiga toxin (Stx)-producing *Escherichia coli* (STEC) are important foodborne pathogens, causing severe illness in humans, including hemorrhagic colitis and hemolytic uremic syndrome (HUS) [1]. STEC isolates from cases of hemorrhagic colitis and/or HUS, or those strains that contain the genes for production of Shiga toxin (Stx), and an adhesin known as intimin, are classified as enterohemorrhagic *E. coli* (EHEC) [2]. The global annual incidence of STEC-related illnesses was recently estimated as 2,801,000 acute illnesses, 3890 cases of HUS, 270 cases of end-stage renal disease, and 230 deaths [3]. Based on data from 2000–2008, the estimated annual incidence of STEC infection in the United States was 175,905 cases, resulting in 2409 hospitalizations and 20 deaths [4]. About 40% of HUS cases stemming from EHEC infections require acute dialysis, and brain involvement is the most frequent cause of acute death [5,6].

EHEC strains cause disease in human patients through a combination of intestinal and extra-intestinal effects [7]. EHEC are thought to infect the human intestine by a mechanism that includes intimate attachment to and effacement of intestinal microvilli [8,9], as was originally demonstrated in a neonatal gnotobiotic piglet model [10,11]. The attaching-and-effacing (A/E) lesions seen in the gnotobiotic piglet [12,13] and other models are dependent upon the production of the outer membrane protein, intimin.

EHEC strains produce either or both of the two main types of Stx, viz., Stx1 and Stx2 [14,15]. These toxins bind to their receptor, viz., globotriaosylceramide (Gb_3_), on the plasma membranes of cells in host tissues, with particular targeting and significance involving the renal microvascular endothelial cells in the human host [7,16]. Stx-mediated injury to endothelial cells results in apoptosis, inflammatory cytokine release, and upregulation of leukocyte adhesion molecules [6,17]. These effects lead to a prothrombotic state resulting in hemorrhage and thrombosis in the tissues of vital organs, especially the kidneys and brain, with development of the HUS and brain infarcts [6]. Central nervous system (CNS) dysfunction is the main cause of acute death in the human patient, and is thought to involve a combination of effects that include Stx-induced vascular injury, endothelial dysfunction, hypertension, and electrolyte disorders [6].

Gnotobiotic piglets have been employed as a model for studying the pathogenesis of EHEC since 1986, when Francis et al. [10] and Tzipori et al. [11] first demonstrated bacterial attachment and microvillous effacement and diarrhea in piglets inoculated with O157:H7 EHEC strain EDL931, originating from a 1982 disease outbreak in Oregon. Tzipori et al. [18] and Francis et al. [19] reported neurological disease in piglets challenged with EHEC strains and collectively demonstrated the presence of hemorrhages, arteriolar necrosis, and infarcts in the brain. Gnotobiotic piglets developed petechial hemorrhages in the cerebellum following inoculation with an isolate of EHEC O157:H7 from a 20-month-old girl that had cerebellar hemorrhages of a very similar appearance [18].

Gnotobiotic piglets also have been used to study the protective effects of passive immunization against Stx with antibodies administered prior to bacterial challenge. The first studies published utilized hyperimmune porcine-origin polyclonal antiserum containing antibodies specific for Stx2 given by the oral [20] or intraperitoneal [21] routes, and in both cases passive immunization protected against brain vascular lesions caused by *E. coli* O157:H7 infection. In another study, hyperimmune porcine-origin polyclonal antiserum containing antibodies specific for Stx2e given via the intraperitoneal route protected conventional weaned pigs against clinical and pathological evidence of disease following an oral challenge of a wild-type Stx2e^+^ *E. coli* porcine isolate [22].

Currently, no approved treatments are available that directly combat or prevent EHEC infection or disease resulting from infection. The therapeutics that have been most extensively developed and tested are monoclonal antibodies (MAb) to Stx1 and Stx2 [23]. Nakao et al. [24] reported the development of a mouse MAb of the immunoglobulin G1 subclass, having *k* light chains that could neutralize the cytotoxic activity of Stx2 and variants derived from patient strains, but not that of variants from animal-derived strains. The Mab, called VTm1.1, was shown to bind to Stx2 B subunits. Subsequently, VTm1.1 was humanized by combining the complementarity-determining regions of VTm1.1 with appropriate human framework and constant regions [25]. In order to further improve binding affinity, several amino acids were changed, which also reduced its potential for stimulating anti-immunoglobulins in humans. The resultant humanized antibody, called TMA-15, retained affinity for Stx2 at a level within two-fold of that of the original murine antibody [25].

Animal experiments have been used to test the efficacy of TMA-15. Kimura et al. [25] demonstrated that TMA-15 prevented death in mice challenged with Stx2. The efficacy of TMA-15 was further demonstrated in a mouse infection model using virulent EHEC O91:H1 Stx2d^+^ strain B2F1, in which case the MAb was administered after bacterial and toxin challenge [26]. In this model, a time-course analysis of the serum Stx2 level showed that the toxin was detectable from 24 h after infection. Protection was demonstrated up to 24 h after infection, but not at 48 h post-challenge. To determine the effective dose, escalating doses were administered at 24 h after infection. Protection of all mice required a TMA-15 dosage of 1.0 mg/kg. The observations of these studies in mice suggest that TMA-15 has potential post-exposure application for the prevention of severe complications associated with EHEC infection. In the present paper, we address this hypothesis using the neonatal gnotobiotic piglet model, because it closely resembles the young human patient (i.e., children) with regard to neurological complications in EHEC O157:H7 infection, the main cause of acute death in patients with HUS.

## 2. Results

### 2.1. Effect of TMA-15 Treatment on Piglet Survival to 192 h after EHEC O157:H7 Inoculation 

Raw data for each piglet and treatment group in the study is provided in Table S1. Regardless of dosage level, a significantly greater proportion of piglets treated with TMA-15 survived for 192 h post-inoculation compared to the placebo-treated piglets (Table 1). The % efficacy of TMA-15 against mortality was 71.4% (Relative Risk = 0.286, *p* <0.05).

**Figure 1:**
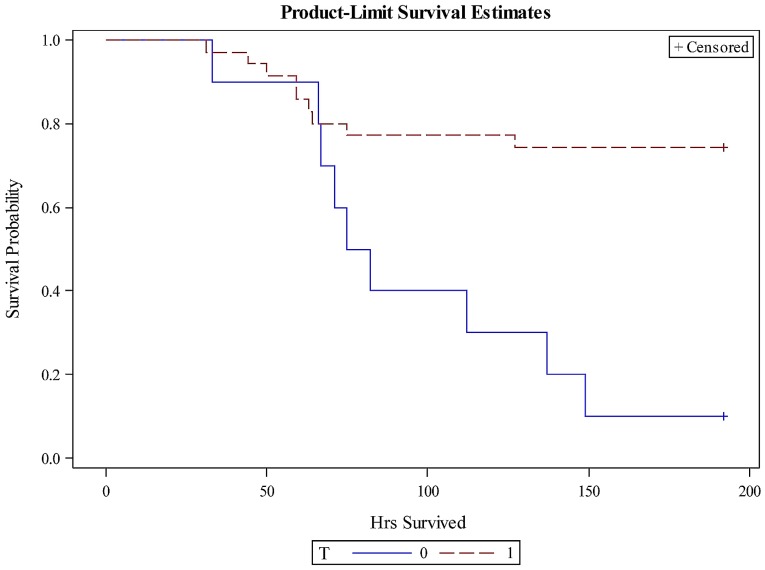
Probability of survival over time in piglets treated with TMA-15 (dashed red line; *n* = 35) or placebo (solid blue line; *n* = 10) 24 h post-challenge inoculation with enterohemorrhagic *E. coli* (EHEC) O157:H7. Survival analysis was conducted using the Kaplan-Meier method (LIFETEST procedure in SAS). + Censored indicates that censored data (piglets surviving to the maximal observation time of 192 h) were used in the analysis.

Overall, piglets treated with TMA-15 survived longer than those treated with the placebo, based on survival analyses (Figure 1). The probabilities of survival over time for the pooled TMA-15 (*n* = 35) and placebo (*n* = 10) groups were significantly different (Chi-square = 8.3496; *p* = 0.0039). The median survival time for the placebo group was 79 h, in contrast to ≥192 h for each of the TMA-15 dosage and pooled dosage groups. The exact mean survival time for each of the TMA-15 groups could not be determined since the sets included censored data.

**Figure 2:**
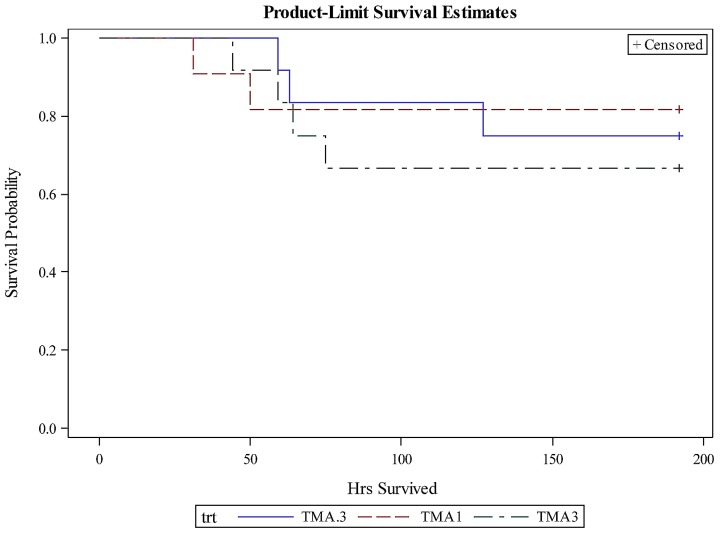
Probability of survival over time in piglets treated with TMA-15 at different dosages [0.3 mg/kg (solid blue line; *n* = 12), 1.0 mg/kg (dashed red line; *n* = 11), and 3.0 mg/kg (dashed green line; *n* = 12)] or with placebo (solid blue line; *n* = 10) 24 h post-challenge inoculation with EHEC O157:H7. Survival analysis was conducted using the Kaplan-Meier method (LIFETEST procedure in SAS). + Censored indicates that censored data (piglets surviving to the maximal observation time of 192 h) were used in the analysis.

Although a significant effect of TMA-15 treatment compared to placebo was detected as noted above, a dosage effect was not detected by survival analysis (Chi-square = 0.4429; *p* = 0.8014; Figure 2).

### 2.2. Effect of TMA-15 Treatment on Weight Change after EHEC O157:H7 Inoculation

The percent weight change in piglets administered TMA-15 was significantly greater than that of piglets administered the placebo after EHEC O157:H7 inoculation; however, a TMA-15 dosage effect was not evident (Table 2). Since weight change was also a function of survival time, the data were normalized as percent weight change per h and analyzed. Similarly, using this parameter, a significant treatment effect for TMA-15 (*p* <0.05), but not a dosage effect was detected.

### 2.3. Relationship between Presence of Brain Lesions and Signs of CNS Dysfunction or Death

Brain lesions consistent with those reported previously in piglets inoculated with EHEC O157:H7 [11,18,20,28] were seen histologically as expected, mainly in piglets with signs of CNS dysfunction. The range of lesions reflected a progression of severity (i.e., from least to most severe): apoptosis of endothelial cells in blood vessels of all types; hemorrhages; thrombosis of blood vessels of all types; vacuolation of neuropil; necrosis of neurons (individual, laminar or confluent); and confluent infarction involving all brain tissue (neurons and neuropil) in one or more layers, e.g., molecular, granular, Purkinje and white matter layers in cerebellum (Figures S1–S8).

The observed frequencies of piglets having lesions as described above in one or more of the five brain section areas and neurological signs (signs of CNS dysfunction) are shown in Table 3. The proportion of piglets with neurological signs concurrent with detectable brain lesions (19/20 (95%)) was significantly greater than the proportion with neurological signs lacking detectable brain lesions [1/20 (5%)] (*p* <0.0001). Hence, the manifestation of neurological signs was strongly associated with, and a predictor of, the presence of brain lesions.

All piglets that died (18/18 (100%)) and 5 of 27 (18.5%) that survived had detectable brain lesions (Table 4).

### 2.4. Relationship between Extent and Severity of Brain Lesions and Death

**Figure 3:**
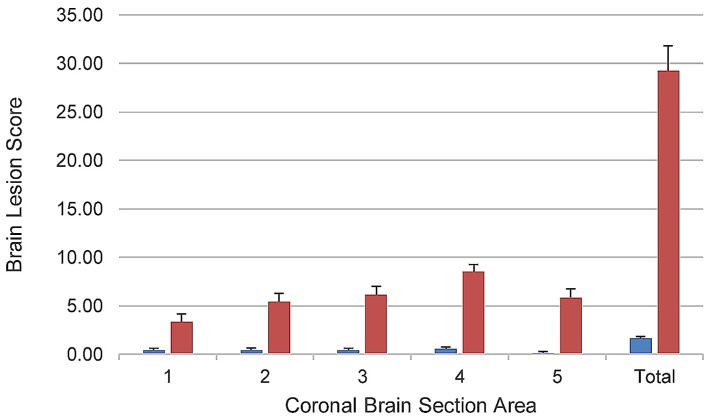
Comparison of brain lesion scores in piglets that were moribund and euthanatized or died (all counted as deaths) during the 192-h post-inoculation period (red bars; *n* = 18) with scores of those that survived for 192 h post-inoculation (blue bars; *n* = 27). Bars show means and standard errors of brain lesion scores (Y axis) as determined by a modification of the brain ischemia scoring scheme of Björkman et al. [29]. Coronal section areas of brain examined (X axis) were the same as those as described by Francis et al. [19] with reference points in parentheses: **1** = medulla oblongata (olivary nucleus); **2** = cerebellum (cerebellar peduncles); **3** = midbrain (corpora quadrigemina); **4** = cerebrum and thalamus (interthalamic adhesion); **5** = cerebrum (genu of corpus callosum); total = sum of scores of all five areas. The mean total lesion score for piglets that survived (2.7 ± 1.6) was significantly lower (*p* <0.001) than the mean score for those that died (29.2 ± 2.7), as determined by Student’s *t* test (TTEST, SAS).

Although death was strongly associated with the presence of brain lesions, since some piglets with brain lesions survived, this suggested that the outcomes of survival or death were directly related to the extent and severity of these lesions. In order to quantify the extent and severity of brain lesions and determine whether there was a quantitative relationship between these two parameters and death in the study, a modified version of the brain ischemia (stroke model) scoring scheme of Björkman et al. [29] was developed and used. This scheme incorporated all lesions related to ischemia and both their extent and severity into one score, allowed for comparison of the extent of involvement of different areas of the brain, and had been previously validated on a piglet brain ischemia model. The scheme was modified by introducing scores for presence of vascular lesions in the absence of neuronal or other brain parenchymal necrosis. Using the modified Björkman scheme, the mean brain lesion score was numerically higher in all five areas of the brain, and the total brain lesion score was significantly higher (*p* <0.0001) in piglets that died compared to those that survived (Figure 3). Hence, a quantitative relationship between brain lesion scores and death was demonstrated using the modified Björkman scheme.

### 2.5. Efficacy of TMA-15 for Prevention and/or Reduction of Extent and Severity of Brain Lesions after EHEC O157:H7 Inoculation

Since a relationship had been established between the extent and severity of brain lesions (as evidenced by modified Björkman scores) and death, the efficacy of TMA-15 on reduction of these lesions was determined. The mean total brain lesion score in piglets treated with TMA-15 (pooled treatment group data) was significantly lower than that of the placebo group (*p* = 0.0255); hence, a significant TMA-15 treatment effect was detected (Table 5). The % efficacy of TMA-15 against brain lesion development was 62.9% (Relative Risk = 0.371; *p* = 0.0004). The mean scores for each of the TMA-15 dosage groups also were significantly lower than that of the placebo group (*p* <0.05), but the scores for the different dosage level groups were not significantly different from each other. Hence, a TMA-15 dosage effect was not detected.

### 2.6. Lack of Effect of TMA-15 Treatment on Diarrhea and Lesions in Organs Other Than the Brain

All piglets in the study developed diarrhea within 24 h post-inoculation with EHEC O157:H7, regardless of treatment. Similarly, all piglets, regardless of treatment, had coliform bacterial colonization of the large intestines in association with classical attaching-effacing (A/E) lesions, and mild to moderate acute multifocal purulent colitis as has been previously reported with EHEC O157:H7 infection. Hence, TMA-15 treatment had no detectable effect on the clinical or pathological parameters of enteric disease caused by EHEC infection, i.e., diarrhea, A/E lesions, and purulent inflammation of the colon.

Lesions other than those seen in the brain and intestines were limited to the liver, lung, and kidney, and seen in a minority of the piglets. Hence, no lesions were seen in the spleens of any of the piglets in the study. Liver lesions were seen in only three piglets, all of which were in Litter 3, and consisted of rare thrombi in the microvasculature. Lung lesions were seen in only three piglets, one each from Litters 1, 3 and 4. Two piglets, one each from Litters 1 and 3, had rare pulmonary thrombi. One piglet from Litter 4 had mild focal acute purulent bronchopneumonia, which may have occurred from aspiration of milk replacer containing bacteria. These sporadic lesions seen were either a part of the natural EHEC infection course (e.g., thrombi in organs) or were circumstantial (e.g., pneumonia in one piglet) and did not affect the outcome of the study.

All piglets in the study, regardless of treatment, had one or more lesions in the kidneys consistent with those previously described with EHEC infection [30,31], but reportedly have not been severe enough to cause statistically significant renal dysfunction [30]. These lesions consisted of rare multifocal glomerular thrombosis, rare multifocal thrombotic or proliferative microangiopathy of afferent arterioles, and rare multifocal apoptosis of tubular epithelial cells (Figures S9–S12). Due to the lack of evidence of causation of clinical effects in previous studies, no attempt was made to conduct clinical pathologic assessments of renal function as a part of the study. Further, since these lesions were present in all piglets of this study and in low numbers, we did not attempt to quantify them. However, we infer that a reduction in the number of renal lesions per piglet would be needed to assess a potential effect of TMA-15 treatment.

### 2.7. Microbiological Tests

Culture of colonic specimens taken from each piglet in the study at necropsy yielded bacteria whose culture and colony characteristics were consistent with that of the inoculum. No contaminants were detected in the colonic contents of piglets in Litters 1–4; however, cultures of all piglets in Litter 5 yielded anaerobes whose colony characteristics were consistent with that of *Clostridium perfringens*. This organism was presumed to have originated from the skin of the sow and entered the isolator units at the time of the cesarean surgery for derivation of Litter 5.

### 2.8. Evaluation of the Potential Effects of Contamination as a Confounder in Litter 5

As with Litters 1–4, no lesions other than those attributable to EHEC O157:H7 infection were detected in the Litter 5 piglets. However, in order to further address the potential for this contaminant to serve as a confounder, all statistical analyses were repeated with only the data from Litters 1–4 (*n* = 35) and the results compared with that of Litters 1–5 (*n* = 45). A reduction in statistical power due to the reduction in the number of observations resulted in an increase in the *p* value for most comparisons. The only result that significantly changed was the effect of TMA-15 treatment on total brain lesion score (Table 6). This was the case because in Litter 5, a clear-cut TMA-15 effect had occurred: both placebo piglets (*n* = 2) had brain lesion scores that fell within the range of the placebo piglets in the other litters, and no brain lesions were detected in any of the TMA-15 piglets (*n* = 8). We concluded that the anaerobic contaminant did not serve as a confounder in the experiment for the following reasons: no lesions were seen other than those attributable to EHEC O157:H7 infection; the placebo piglets were not protected from brain lesions; and, the clinical outcomes and their rate of occurrence did not differ from that of Litters 1–4.

## 3. Discussion

The results of this study demonstrate that the humanized anti-Stx2 monoclonal antibody TMA-15 is highly protective against neuropathological sequelae of EHEC infection in the neonatal gnotobiotic piglet model when administered within 24 h after the onset of infection. This study extends the observations made by Yamagami et al. [26] who reported that TMA-15 increased the survival of mice when administered 24 h after challenge with 10^10^ CFU EHEC. These results also extend those of Mukherjee et al. [32] and Sheoran et al. [33] who reported that an anti-Stx2 MAb increased the survival of gnotobiotic piglets after EHEC inoculation. The studies by Mukherjee et al. [32] and Sheoran et al. [33] differed from the present one in several ways: type of MAb and specific one used; MAb dosage levels; EHEC O157:H7 challenge strain used; EHEC inoculum level; length of observation period after challenge; range of response variables monitored; response variables subjected to statistical hypothesis testing (Table 7). The present study addressed three MAb dosage levels and one time of administration, but provided a more extensive analysis of its efficacy against brain lesions, with the development and implementation of a relevant brain lesion scoring scheme, and statistical hypothesis testing of all main response variables.

In the present study, infarcts and vascular lesions, including arteriolar necrosis were seen in all five coronal section areas of brain examined, and TMA-15 was demonstrably effective for the prevention or reduction of these lesions. In addition, the study addressed whether Stx2-associated kidney lesions as had been previously reported [30,31] were present. Renal lesions as previously reported were seen in all piglets, but the effect of TMA-15 on their extent was difficult to evaluate since these lesions were low in number, yet were seen in all piglets, regardless of their clinical outcome or brain involvement. Although piglets infected with EHEC O157:H7, including those with neurological signs and brain lesions have thrombotic microangiopathy and other lesions shown to be due to Stx2 [30,31], these piglets do not have significantly different serum chemistry values that constitute evidence of renal uremia [30]. Consistent with this conclusion, earlier studies involving gnotobiotic piglets inoculated with EHEC O157:H7, including those that developed neurological signs and brain lesions as seen in the present study, had no evidence of renal compromise based on serum chemistries [34].

The pig is an excellent model for human brain anatomy and pathophysiology, and in this regard has frequently been used to study the effects of hypoxia and ischemia [35]. The advantages of the gnotobiotic piglet as a model to study Stx-induced systemic lesions and disease effects in EHEC infection, in particular those affecting the brain, have further been noted [36,37,38]. It is especially important to note that a significant proportion of children [18,39,40,41,42], as opposed to adults [43] dying from HUS, most of which are due or thought to be due to EHEC infection, develop hemorrhages, microangiopathy, and infarcts in the brain. The vascular and ischemic lesions (microangiopathy and infarcts) in these patients closely resemble those seen in the gnotobiotic piglet model, as in the present study and previously reported. Hence, a determination of the efficacy of an intervention such as TMA-15 for the prevention or reduction of these lesions, and usage of an appropriate pathology scoring scheme is highly warranted. Weaknesses of the neonatal gnotobiotic piglet EHEC O157:H7 infection model are that the piglets do not develop: (1) hemolytic anemia; (2) thrombocytopenia; (3) acute kidney injury or consistent lesions that result in azotemia or uremia; (4) a high incidence of bloody diarrhea or hemorrhagic colitis [28,30].

EDL933 was used as the challenge strain because it has a high level of virulence in gnotobiotic pigs, and with a high degree of regularity causes CNS disease and brain lesions in these animals [19,28]. In previous neonatal gnotobiotic piglet studies it reliably induced brain lesions and, although it produces both Stx1 and Stx2, CNS signs and brain lesions were associated with Stx2 but not Stx1 [19]. A derivative of EDL933 that lost the Stx2-converting phage but retained the Stx1-converting phage (strain 933D) did not cause CNS signs or brain lesions in the neonatal gnotobiotic piglet EHEC O157:H7 enteric infection model [19]. In a subsequent large-scale study, using 100 gnotobiotic piglets and 20 EHEC O157:H7 strains, the same conclusions were drawn. The amount of Stx2 produced by the strain was correlated with a reduction in survival, manifestation of CNS signs, and brain infarcts [28]. In that same study, the amount of Stx1 produced by the strain was not correlated with length of survival, CNS signs or brain infarcts. In a study by another group, gnotobiotic piglets were inoculated with isogenic Stx1^+^ and Stx2^+^ deletion mutants of EDL933 [44]. CNS signs were only seen with the wild-type parent (Stx1^+^, Stx2^+^) and Stx2^+^-only mutant derivatives. One of six piglets inoculated with an Stx1^+^ -only mutant derivative had two discrete focal lesions in the brain, but no CNS signs. Hence, Stx1 production by EDL933 in enteric infection of neonatal gnotobiotic piglets has very rarely been associated with CNS lesions and, to our knowledge, yet been shown to cause CNS signs. For these reasons, we did not consider production of Stx1 by EDL933 to be a confounder in the present study. However, it should be noted that Stx1 administered parenterally in relatively large doses to the neonatal gnotobiotic piglet can result in development of the same brain lesions as those attributed to Stx2 in the enteric bacterial infection model [45]. Hence, the Stx serum concentration that occurs in the infection would appear to be a key.

In conclusion, this study demonstrated that urtoxazumab (TMA-15), a humanized monoclonal antibody specific for Stx2 and previously shown to be safe in human patients, provides significant protection against brain lesions, neurological signs, and death resulting from brain involvement when administered within 24 h after the onset of EHEC infection in the neonatal gnotobiotic piglet model. These results suggest that TMA-15 may potentially prevent or reduce vascular necrosis and infarction of the brain attributable to Stx2 in human patients acutely infected with EHEC. Since CNS disease in the gnotobiotic piglet following enteric infection with EHEC O157:H7 is predominantly, if not exclusively, the result of toxic effects of Stx2 and TMA-15 is specific for Stx2, we infer that protection in the present study was the result of Stx2 neutralization. We do not infer that TMA-15 treatment will result in complete protection against systemic effects in human patients resulting from enteric infections with EHEC O157:H7 strains that produce both Stx1 and Stx2. Further studies with TMA-15 to define the effects of dosage and time of treatment in relation to onset of EHEC infection are warranted.

## 4. Materials and Methods

### 4.1. Monoclonal Anti-Stx2 Antibody and Placebo Control

TMA-15, a humanized mouse MAb against Stx2, was prepared as described elsewhere [25], and provided by Teijin LLC in sealed ampules (Lot Numbers TEIn004 and TEIs004) which were received on 12 October 2008. The ampules, each containing 5 mL TMA-15 at a concentration of 4 mg/mL, were stored at 4 °C and opened immediately before use. Prior studies conducted at Teijin LLC had determined that TMA-15 was stable for ≥2 years. At the time of use, TMA-15 was diluted to the desired concentration (0.3, 1.0 or 3.0 mg/kg body weight) with physiological saline. Placebo (MAb diluent) was also provided by Teijin LLC in sealed ampules, stored under the same conditions as TMA-15, and used without dilution. The placebo and TMA-15 diluent were an aqueous solution (pH 6.0) consisting of 20 mM sodium citrate, 60 mM sodium chloride, 4% *w*/*v* sucrose, and 0.01% *w*/*v* Polysorbate 80. Both TMA-15 and placebo were obtained from ampules using a filter needle.

### 4.2. Ethical Statement and Animal Care

The care and use of animals for these experiments was approved by the South Dakota State University Institutional Animal Care and Use Committee under assurance number #A3958-01 and approval number 06-A014 (23 March 2006 with modification on 25 September 2008). Animal experiments began on 16 January 2009 and ended on 8 August 2009. Late term pregnant sows were purchased from local commercial producers and subjected to cesarean section for the delivery of gnotobiotic piglets on the 112th day of gestation (with full term being 114 days in pigs). Serological tests were performed to confirm that the sows had not been exposed to porcine reproductive and respiratory syndrome virus [46] or porcine parvovirus [47], either of which could cause transplacental infection and complicating disease effects. Cesarean section surgeries were conducted under aseptic conditions by methods previously described [48]. Piglets were reared in stainless steel isolators fitted with flexible plastic canopies with two sets of sleeve-attached gloves for animal and supply handling. Each isolator housed up to 4 piglets, with each animal in an individual pen. Isolator rooms were maintained in an ambient temperature of 33 °C, which is optimal for rearing neonatal piglets. Piglets were fed a sterile, commercial milk replacer (EsbiLac, PetAg, Ihync, Hamilton, IL, USA) three times daily, gradually increased in amount to accommodate for the growth requirements of the animals.

### 4.3. Administration of TMA-15 and Placebo Control

TMA-15 and placebo were administered by bolus injection into the peritoneal cavity using a disposable syringe and needle at a dose volume of 2.5 mL/kg BW, and were given only once per piglet at 24 h after EHEC O157:H7 strain EDL933 inoculation. A preliminary study confirming that TMA-15 is absorbed into the bloodstream of neonatal gnotobiotic piglets following intraperitoneal injection and establishes high anti-Stx 2 titers, including 24 h post-inoculation with EHEC O157:H7 24 h, was conducted with other litters of gnotobiotic piglets prior to the present study. In that study, 19 piglets originating from 2 litters were divided at random and administered TMA-15 at a dosage of 3.0 mg/kg BW (*n* = 15) or placebo (*n* = 4) (Table S2). Anti-Stx2 titers were determined at varying time intervals up to 192 h (Table S2). Anti-Stx2 titers were determined with a validated ELISA with a lower limit of quantification of 100 ng/mL (MDS Pharma Service, Inc., St. Laurent, QB, Canada). High serum anti-Stx2 titers were maintained through a 168 h post-TMA-15 administration period, which in turn had been given 24 h post-inoculation with EHEC O157:H7 (Table S3, Figures S13 and S14). In this study, 10 of 15 piglets treated with TMA-15 at a dosage of 3.0 mg/kg survived (66.7%), compared to 0 of 4 placebo piglets.

### 4.4. Bacterial Strain and Inoculum

*Escherichia coli* strain EDL933 (O157:H7; Stx1, Stx2; ATCC® 43895) originally obtained from Centers for Disease Control and Prevention (CDC), Atlanta, Georgia, and stored in liquid nitrogen, was used in this study. EDL933 was used as the challenge strain because it has a high level of virulence in gnotobiotic pigs, and with a high degree of regularity causes CNS disease and brain lesions in these animals [19,28]. The bacterium was inoculated in BBL trypticase soy broth (TSB, Becton, Dickinson, Sparks, MD, USA) and incubated for 12–18 h at 37 °C. Piglets received 3 mL of the inoculated medium which contained approximately 3 × 10^9^ colony forming units (CFU), and was delivered per os 22–24 h after birth along with a milk replacer meal.

An additional preliminary study (separate from the study described in Section 4.3) to determine the peripheral blood concentrations of Stx2 over time after oral inoculation of EHEC O157:H7 strain EDL933 at an inoculum level of 3 × 10^9^ CFU in 22–24 h-old gnotobiotic piglets was conducted with other litters under the same conditions and methodologies prior to the present study. Twenty-nine gnotobiotic piglets originating from 3 litters were inoculated as described above with EDL933 and jugular vein blood samples were obtained, with none of these piglets having been treated with placebo or TMA-15 (Table S4). Only 2 of 29 piglets survived the 192-h observation period (Table 2) and 1 of these had CNS signs at 192 h post-inoculation. All 27 piglets that died before 192-h post-inoculation, displayed CNS signs.

Blood samples from these piglets were centrifuged immediately after removal from the isolator units, frozen at −76 °C, and then shipped on dry ice to SRL Medisearch, Inc. (Tokyo, Japan), Sample Measurement Section, Hachioji 3rd Laboratory, 153 Komiya-cho, Hachioji, Tokyo 192-0031, Japan. At this laboratory, the Stx2 serum concentrations were determined by an ELISA that had been validated for piglet serum samples with the lower limit of quantification (LLOQ) determined to be 10 pg/mL.

Individual serum concentration curves are shown in Figure S15. Stx2 was detected within 12 h post-inoculation in 8 of 13 piglets, indicating that Stx2 had entered the blood circulation in a short period after EDL933 inoculation. Serum Stx2 concentrations increased in a time-dependent manner from the first to the second scheduled bleeding in all piglets, and until the terminal time point in 16 of 23 piglets. The other 7 piglets slowed a slight decrease or no change from the second bleeding to bleeding at euthanasia. The relationship between terminal time point and terminal Stx2 serum concentration in each piglet is shown in Figure S16. Serum Stx2 titers varied substantially from piglet to piglet, such that no statistically significant trends could be established predicting a lethal endpoint, although the data did confirm the presence of Stx2 in the blood of inoculated piglets in the absence of TMA-15 treatment, and a trend for an increase in Stx2 serum concentrations over time for at least the first 72 h after inoculation (the limit of sampling other than immediately prior to death).

### 4.5. Piglet Observation and Sample Collection

Subsequent to inoculation, piglets were clinically monitored every 4 h until signs of CNS disease appeared, after which the affected piglets were monitored every 2 h. Clinical signs monitored included: diarrhea, weight loss, dehydration (eyes sunken in orbits), lethargy, anorexia, ataxia, head tilt or circling, nystagmus, inability to stand, lateral recumbency, moribund state (see definition, below), opisthotonus, and tonic-clonic convulsions (“paddling”). Piglets were weighed at the time of TMA-15 inoculation and again at the time of euthanasia, before necropsy. Infected animals judged by the examiner to be moribund were immediately subjected to euthanasia. For the purposes of this study, the moribund state was defined as irreversible lateral recumbency (which also occurred in some cases concurrent with opisthotonus and convulsions) and/or extended anorexia (missing 2 consecutive meals). Animals that survived (i.e., did not become moribund) were euthanatized 192 h post-challenge at the termination of the observation period. Euthanasia was accomplished by intramuscular injection of Telazol® (Fort Dodge, IA, USA) at a dosage of 6 mg/kg body weight (3.0 mg/kg tiletamine and 3.0 mg/kg zolazepam) to induce unconsciousness and general anesthesia [49] followed by electrocution. This protocol was in accordance with the AVMA Guidelines on Euthanasia [50], and approved by the South Dakota State University Institutional Animal Care and Use Committee.

At the time of death, body weights were recorded and piglets were subjected to necropsy and specimen collection. Specimens were collected for histopathological examination, which included: brain, ileum, cecum, colon, rectum, liver, lung, spleen, and kidney. These specimens were fixed in 10% neutral buffered formalin, and subsequently processed, sectioned, placed on glass microscope slides, and stained with hematoxylin and eosin for histopathological examination by standard procedures. Prior to tissue processing, formalin-fixed brains were grossly coronally-sectioned to allow for examination of tissue in the following areas (anatomic reference points): medulla oblongata (olivary nucleus); cerebellum with medulla oblongata (cerebellar pedunucles); midbrain (corpora quadrigemina); and two levels of cerebrum (interthalamic adhesion and genu of corpus callosum), as previously described [19].

In addition to the tissues for histopathology, a fresh, unfixed segment of the colon was collected at the time of necropsy for bacterial culture to test for presence of the inoculated strain, and to assess for potential contamination. Colonic contents were obtained aseptically from the specimens and subjected to direct culture at 37 °C on 5% sheep blood agar in trypticase soy agar base (Difco Laboratories, Detroit, MI, USA) in an aerobic environment for 18 h, or in an anaerobic chamber for 48 h. After incubation, cultures were examined for organisms whose colony or cultural characteristics were consistent or inconsistent with the inoculum.

### 4.6. Experimental Objective and Design

The overall goal of this work was to evaluate the efficacy of urtoxazumab (TMA-15) for prevention of neurological complications of EHEC O157:H7 infection using the neonatal gnotobiotic piglet model. A total of 45 piglets originating from 5 litters were used in the study with piglets in each litter selected at random for assignment to treatment groups. To control for litter variability, each litter was divided such that 2 piglets at random were assigned to the placebo group, and 2 or more piglets at random were assigned to each TMA-15 dosage group (Table 8). If the litter contained an odd number of piglets, the extra piglets were placed at random into 1 or more TMA-15 dosage groups. If the litter contained only 4 piglets, the 2 assigned at random to TMA-15 were placed into the 3.0 mg/kg group.

### 4.7. Histological Evaluation of Tissues for Microscopic Lesions

Sections of tissues that were collected at necropsy and processed by routine methods for histopathology were examined by one of the authors (R.A.M.) who was blinded to the treatment group. Brain tissues were scored from 0–12 for the degree of injury using a modified version of the scheme described by Björkman et al. [29] (Table 9). The scheme was based on four categories of morphological changes, which were cumulative and of increasing severity. The first category consisted of necrosis of vascular intimal and/or medial cells with thrombosis and hemorrhage (grade 1–3) and no evidence of neuronal or other brain parenchymal necrosis. The second was necrosis of individual neurons (grade 4–6). The third was necrosis of a group or layer of neurons, termed laminar necrosis (grade 7–9). The fourth was necrosis of all cells within a defined area, and was referred to as a confluent necrosis (grade 10–12). Variations in scores within these categories reflected the extent of the lesions within a brain section. Total neuropathology scores were calculated as the sum of the scores from all five brain section areas.

Following routine histopathological examination for any type of abnormality, sections of other tissues were in particular noted for lesions related to EHEC infection. Sections of intestinal tissues (jejunum, ileum, cecum, spiral colon, and rectum) were examined and noted for presence of coliform bacteria, A/E lesions, and inflammatory changes consistent with EHEC infection. Kidney tissue sections were examined and noted for presence of hemorrhage, thrombosis, microangiopathy, and tubular epithelial necrosis and apoptosis. Sections of liver, spleen, and lung were examined and noted in particular for presence of thrombotic and inflammatory lesions.

### 4.8. Statistical Analysis

Statistical analyses were conducted using SAS version 9.4 (SAS Institute, Cary, NC, USA). Statistically significant results were represented by values of *p* <0.05. For those variables where there were but two treatments, a *t*-test (with Satterthwaite’s correction for degrees of freedom when heteroscedasticity was present) was run with a Wilcoxon Rank Sum non-parametric test to confirm the results. In all cases, the two tests indicated similar results. For more than two treatments the usual analysis of variance with the non-parametric equivalent (Kruskal Wallis test) was run. For two-factor experiments a two-way analysis of variance was run, in which case only the treatment was significant and never the factor “time” or the interaction. For correlations, both the Pearson and non-parametric Spearman’s correlation were conducted with similar results. The LIFETEST procedure with adjustment for censored data was used to compute survival curves (nonparametric estimates of the survivor function) by the product-limit (Kaplan-Meier) method.

## Figures and Tables

**Table 1:** Proportion of piglets by treatment group that survived for 192 h after inoculation with enterohemorrhagic *E. coli* (EHEC) O157:H7 *.

Treatment	Litter					Total (%) ^**^
	1	2	3	4	5	
Placebo	0/2	0/2	0/2	0/2	1/2	1/10 (10.0) ^a^
TMA-15, 0.3 mg/kg ^†^	0/2	NT ^‡^	2/3	4/4	3/3	9/12 (75.0) ^b^
TMA-15, 1.0 mg/kg	0/1	NT	3/4	3/3	3/3	9/11 (81.8) ^b^
TMA-15, 3.0 mg/kg	0/2	2/2	1/3	3/3	2/2	8/12 (66.7) ^b^
Pooled TMA-15 data	0/5	2/2	6/10	10/10	8/8	25/35 (71.4) ^b^

* Number survived/number inoculated. ** Total proportion for all 5 litters combined and expressed as a percentage. ^†^ Dosage of TMA-15 in mg/kg body weight, administered intraperitoneally 24 h post-inoculation. ^‡^ NT, not tested since the litter contained only 4 piglets, and the a priori study design was to include a minimum of 2 piglets per treatment group per litter. ^a,b^ Unlike superscripts denote significantly different proportions (*p* < 0.05) based on Chi-squared and Fisher’s exact test analyses (FREQ Procedure, SAS) which gave similar results.

**Table 2:** Mean weight change and weight change per h in piglets after inoculation with EHEC O157:H7.

Treatment	% Weight Change *	% Weight Change/h *	Difference in % Weight Change and % Weight Change/h (in Parentheses) from External Non-Toxin Control ^†^
Placebo (*n* = 10)	5.8 ± 3.5 ^a^	0.04 ± 0.02 ^a^	−22.3 ± 1.4 (−0.11 ± 0.1)
TMA-15, 0.3 mg/kg (*n* =12)	24.8 ± 4.9 ^b^	0.13 ± 0.03 ^b^	−3.3 ± 1.5 (−0.02 ± 0.1)
TMA-15, 1.0 mg/kg (*n* = 11)	30.4 ± 5.0 ^b^	0.15 ± 0.03 ^b^	2.3 ± 1.6 (0.00 ± 0.1)
TMA-15, 3.0 mg/kg (*n* = 12)	25.7 ± 6.5 ^b^	0.13 ± 0.04 ^b^	−2.4 ± 1.7 (−0.02 ± 0.1)
Pooled TMA-15 data (*n* = 35)	26.9 ± 3.1 ^b^	0.14 ± 0.02 ^b^	1.2 ± 1.2 (−0.01 ± 0.72)
External non-toxin control (*n* = 2) ^†^	28.1 ± 1.31 ^b^	0.15 ± 0.01 ^b^	NA

* Mean ± standard error of mean. % weight change represents the difference in body weight occurring between the times of administration of treatment (TMA-15 or placebo) and euthanasia or necropsy, if death had already occurred, expressed as a percentage. % weight change per h represents weight change as previously defined divided by the number of h represented by the time differential, expressed as a percentage. Means for placebo and pooled TMA-15 groups were compared using Student’s *t* test (SAS TTEST). Additionally, means for placebo and the 3 dosage levels of TMA-15 were compared using the GLM (General Linear Model) procedure, LS (Least Squares) Means in SAS. **^†^** Since this study did not include non-inoculated piglets, historical data from age-matched gnotobiotic piglet controls from another study [27] was used to estimate the extent to which TMA-15 treatment normalized the weight change. These controls (*n* = 2) were weighed at the same ages as in the present study (at 1 and 9 days of age, 192 h apart) prior to inoculation and were raised under comparable conditions (type of milk replacer, frequency, amount, isolator units, etc.). The % weight change and % weight change per h for the external controls was 28.1 ± 1.31 and 0.15 ± 0.01, respectively. The differences between the means were determined by simple subtraction, whereas that for SEMs was calculated by the following formula: SEM difference = SQRT ([SD group 1/*n*1] + [SD group 2/*n*2]), where SQRT = square root, SD = standard deviation, and *n* = number of piglets in group. ^a,b^ Unlike superscripts denote significantly different means (*p* < 0.05). NA = not applicable.

**Table 3:** Contingency table (2 × 2) comparing numbers of piglets with brain lesions and neurological signs.

Outcome		Brain Lesions		Total
Neurological Signs	Absence (0) or Presence (1)	0	1	
	0	21	4	25
	1	1	19 *	20
Total		22	23	45

* The proportion of piglets manifesting neurological signs that had detectable brain lesions [19/20 (95%)] was significantly greater than the proportion manifesting neurological signs that lacked detectable brain lesions (1/20 (5%)) (*p* <0.0001), as determined by Fisher’s exact test (FREQ Procedure, SAS).

**Table 4:** Contingency table (2 × 2) comparing numbers of piglets with brain lesions and death.

Outcome		Brain Lesions		Total
Death	Absence (0) or Presence (1)	0	1	
	0	22	5	27
	1	0	18 *	18
Total		22	23	45

* The proportion of piglets that died that had detectable brain lesions (18/18 (100%)) was significantly greater than the proportion that died that lacked detectable brain lesions (0/18 (0%)) (*p* <0.0001), as determined by Fisher’s exact test (FREQ Procedure, SAS).

**Table 5:** Mean total brain lesion scores in piglets after inoculation with EHEC O157:H7.

Treatment	Total Brain Lesion Score *
Placebo (*n* = 10)	27.5 ± 3.6^a^
TMA-15, 0.3 mg/kg (*n* =12)	11.8 ± 5.2^b^
TMA-15, 1.0 mg/kg (*n* = 11)	4.7 ± 2.8^b^
TMA-15, 3.0 mg/kg (*n* = 12)	10.8 ± 4.8^b^
Pooled TMA-15 groups (*n* = 35)	9.3 ± 2.6^b^

* Mean ± standard error of mean. % weight change represents the difference in body weight occurring between the times of administration of treatment (TMA-15 or placebo) and euthanasia or necropsy, if death had already occurred, expressed as a percentage. % weight change per h represents weight change as previously defined divided by the number of h represented by the time differential, expressed as a percentage. Means for placebo and pooled TMA-15 groups were compared using Student’s *t* test (SAS TTEST). Additionally, means for placebo and the 3 dosage levels of TMA-15 were compared using the GLM (General Linear Model) procedure, LS (Least Squares) Means in SAS. ^a,b^ Unlike superscripts denote significantly different means (*p* < 0.05).

**Table 6:** Comparison of P values with and without inclusion of Litter 5 data *.

Response Variable	Comparison	Test	Litters 1–5 (*n* = 45)	Litters 1–4 (*n* = 35)
Survival	Across P and 3 dosages of T	Chi-Square	0.0327	0.1362
Survival	P versus pooled T	Chi-Square	0.0039	0.0210
Survival curve (survival over time)	P versus pooled T	Kaplan-Meier	0.0165	0.0658
Weight change	P versus pooled T	*t* test	0.0014	0.0038
Weight change/h	P versus pooled T	*t* test	0.0073	0.0132
Brain lesions and neurological signs	Relationship	Fisher’s exact test	<0.0001	<0.0001
Brain lesions and death	Relationship	Fisher’s exact test	<0.0001	<0.0001
Total brain lesion score	S versus D	*t* test	<0.0001	<0.0001
Total brain lesion score	P versus T	*t* test	0.0010	0.0440
% Efficacy against death ^†^	P versus T	1-Relative Risk	<0.0004	<0.001
% Efficacy against brain lesions ^‡^	P versus T	1-Relative Risk	<0.0004	<0.0086

* *p* = placebo-treated; T = TMA-15-treated; pooled T = data from 3 dosage groups pooled; S = survived; D = died (includes moribund piglets that were euthanatized); *t* test = Student’s *t* test. ^†^ The % efficacy of TMA-15 against death for *n* = 45 was 71.4%, compared to 66.7% for *n* = 35. ^‡^ The % efficacy of TMA-15 against brain lesions for *n* = 45 was 62.9%, compared to 51.9% for *n* = 35.

**Table 7:** Comparison of studies evaluating the efficacy of anti-Stx2 monoclonal therapy to prevent systemic complications following intestinal infection with EHEC O157:H7 in gnotobiotic piglets.

Parameter	Present Study	Reference [32]	Reference [33]
Monoclonal antibody type	Humanized	Human	Human
Monoclonal antibody (isotype)	TMA-15 (IgG1κ)	3E9, 5H8, 2F10, 5C12 (IgG1κ)	5C12 (IgG1κ)
Monoclonal antibody Stx subunit specificity	B	A, B, A, A *	A
Monoclonal antibody dosage (h post-inoculation)	0.3, 1.0 or 3.0 mg/kg (24)	3.0 mg (6 or 12)	1.5 or 3.0 mg/kg (24)
			0.05, 0.1, 0.2, 0.4, 0.75, 3.0 or 6.0 mg/kg (48)
EHEC inoculum strain (Stx type)	EDL933 (Stx1^+^, Stx2^+^)	86-24 (Stx2^+^)	86–24 (Stx2^+^)
EHEC inoculum level	~3 × 10^9^ CFU ^†^	~1 × 10^1^° CFU	~1 × 10^10^ CFU
Age of pig in hours at time of EHEC inoculation	24	24	24
Maximum number of days of observation after inoculation	8	10	14
Fluid therapy used to control dehydration after inoculation	No	No	Yes
Response variables monitored	Diarrhea, anorexia, lethargy, anorexia, dehydration, weight change, weight change per h, CNS signs, moribund state, survival, brain lesions (5 levels), brain lesion score (5 levels)	Diarrhea, dehydration, CNS signs, brain lesions (2 levels: cerebral cortex and cerebellum), survival, serum human IgG concentration	Diarrhea, anorexia, depression, dehydration, weight loss, brain lesions (2 levels: cerebral cortex and cerebellum), survival, serum human IgG1(κ) concentration
Response variables statistically analyzed by hypothesis testing	Weight change, weight change per h, CNS signs, survival, brain lesions (5 levels), brain lesion score (5 levels)	Survival	Not indicated

* Order of listing of Shiga toxin subunit specificities refers to respective order of monoclonal antibodies listed in row above. ^†^ CFU, colony-forming units.

**Table 8:** Number of piglets used in the study.

Litter	Placebo	TMA-15 Dosage (mg/kg Body Weight)			Total
		0.3	1.0	3.0	
1	2	2	1	2	7
2	2	0	0	2	4
3	2	3	4	3	12
4	2	4	3	3	12
5	2	3	3	2	10
Total	10	12	11	12	45

**Table 9:** Neuropathological scoring scheme.

Neuropathological Score	Percentage of Area Affected	Morphological Changes
0	0	No injury
1	<20	Vascular necrosis with thrombosis and hemorrhage
2	20–50	
3	>50	
4	<20	Neuronal necrosis
5	20–50	
6	>50	
7	<20	Laminar necrosis
8	20–50	
9	>50	
10	<20	Confluent necrosis
11	20–50	
12	>50	

## Supplementary Materials

The following are available online at www.mdpi.com/2072-6651/9/2/49/s1, Figure S1. Low-magnification photomicrograph of cerebrum of placebo control piglet (No. 2F-1D) that was euthanatized when it became moribund 67 h post-inoculation with EHEC O157:H7 strain EDL933. Three foci of infarction (outlined by rectangles) are seen, which at this magnification appear as areas of pallor. Bar in lower right corner = 1 mm. Original objective magnification = 2x. Hematoxylin and eosin stain. Figure S2. Higher magnification of area within box no. 3 in previous photomicrograph. Area of pallor includes vacuolated neuropil and shrunken, pyknotic cells. Borders of most severely affected area are denoted by arrows. Area in lower left corner below the arrows is also affected, perhaps with less severe neuropil vacuolation. Bar in lower right corner = 200 μm. Original objective magnification = 10x. Hematoxylin and eosin stain. Figure S3. Higher magnification of area within infarct is shown in previous photomicrograph. Neuron No. 1 is necrotic, as evidenced by being shrunken with pyknotic nucleus and eosinophilic perikaryon. Neuron No. 2 is also necrotic, as evidenced by being shrunken with karyolytic nucleus and eosinophilic perikaryon. Other neurons in the figure (unlabeled) are necrotic. Neuropil is pale-staining and vacuolated. Bar in lower right corner = 20 μm. Original objective magnification = 60x. Hematoxylin and eosin stain. Figure S4. Photomicrograph of medulla oblongata of placebo control piglet (no. 2F1B) that was euthanatized when it became moribund 33 h after inoculation with EHEC O157:H7 strain EDL933. A fibrin thrombus (horizontal arrow with label) with entrapped red blood cells occluding a venule is seen in the center of the field. Fibrin strands are seen within a perivascular space (vertical arrow with label). Bar in lower right corner = 20 μm. Original objective magnification = 60x. Hematoxylin and eosin stain. Figure S5. Photomicrograph of medulla oblongata is shown in previous figure. Endothelial cells within a venule show the morphologic hallmarks of apoptosis (arrows with label). Bar in lower right corner = 20 μm. Original objective magnification = 60x. Hematoxylin and eosin stain. Figure S6. Photomicrograph of cerebellum of piglet (No. 3F1C) treated with TMA-15 (3.0 mg/kg) 24 h post-inoculation with EHEC O157:H7 strain EDL933. This piglet was the only one in the treatment group that became moribund (75 h post-inoculation). Lesions seen in this section include multiple coalescing infarcts (confluent necrosis) involving the molecular, granular, Purkinje and white matter layers, and associated hemorrhage. Bar in lower right corner = 100 μm. Original objective magnification = 20x. Hematoxylin and eosin stain. Figure S7. Photomicrograph of mesencephalon of placebo control piglet (No. 2F1B). A ring-shaped area of hemorrhage caused by circumferential bleeding around a necrotic arteriole is seen in the white matter in the center of the field. The material in the center of the lesion (arrow) is fibrin. Bar in lower right corner = 50 μm. Original objective magnification = 40X. Hematoxylin and eosin stain. Figure S8. Photomicrograph of thalamus from same piglet is shown in previous figure. A thrombus (arrow) is seen in a venule in the lower left corner. The brain parenchyma immediately surrounding the thrombosed vessel is infarcted, as evidenced by necrotic neurons and other cells, and vacuolated neuropil. Bar in lower right corner = 50 μm. Original objective magnification = 40x. Hematoxylin and eosin stain. Figure S9. Photomicrograph of kidney of piglet 2F1C which was treated with TMA-15 at a dosage of 3 mg/kg BW and survived to 192 h post-inoculation with EHEC O157:H7 strain EDL933. Glomerulus in center of field contains capillary thrombi (horizontal arrows), and is hemorrhagic (vertical arrow). This piglet did not develop signs of CNS dysfunction and had no detectable brain lesions. Bar in lower right corner = 20 μm. Original objective magnification = 60x. Hematoxylin and eosin stain. Figure S10. Photomicrograph of kidney of piglet 3F2D which was treated with TMA-15 at a dosage of 0.3 mg/kg BW and survived to 192 h post-inoculation with EHEC O157:H7 strain EDL933. Two arterioles in the center of the field (vertical arrows) have undergone proliferative microangiopathy, as evidenced by intimal proliferation that has thickened the walls and obliterated the lumens of these vessels. In addition, some myocytes in the tunica media have undergone fibrinoid change or coagulative necrosis. This piglet did not develop signs of CNS dysfunction and had no detectable brain lesions. Bar in lower right corner = 20 μm. Original objective magnification = 60x. Hematoxylin and eosin stain. Figure S11. Photomicrograph of kidney of piglet 3F3D which was treated with TMA-15 at a dosage of 1.0 mg/kg BW and survived to 192 h post-inoculation with EHEC O157:H7 strain EDL933. An arteriole in the center of the field (vertical arrow) has undergone thrombotic microangiopathy with complete obliteration its lumen. Some myocytes in the tunica media have undergone fibrinoid change or coagulative necrosis with karyorrhexis and karyolysis of nuclei. This piglet did not develop signs of CNS dysfunction and had no detectable brain lesions. Bar in lower right corner = 20 μm. Original objective magnification = 60X. Hematoxylin and eosin stain. Figure S12. Photomicrograph of kidney of piglet 2F1B which was treated with placebo and died 67 h post-inoculation with EHEC O157:H7 strain EDL933. A renal tubule epithelial cell in the center of the field has undergone apoptosis, as evidenced by the presence of bleb fragments (arrows). Bar in lower left corner = 20 μm. Original objective magnification = 60x. Hematoxylin and eosin stain. Figure S13. Mean serum TMA-15 concentrations (ng/ml) over time in neonatal gnotobiotic piglets inoculated with EHEC O157:H7 and treated by intraperitoneal injection with TMA-15 (3.0 mg/kg) 24 h after bacterial inoculation. Figure S14. Serum TMA-15 concentrations (ng/ml) over time in individual neonatal gnotobiotic piglets inoculated with EHEC O157:H7 and treated by intraperitoneal injection with TMA-15 (3.0 mg/kg) 24 h after bacterial inoculation. Key to piglet ID is shown in Table S3. Figure S15. Serum Stx2 concentrations over time in individual neonatal gnotobiotic piglets after oral inoculation with EHEC O157:H7 strain EDL933 at 22–24 h of age. The key in the upper left hand corner shows the identification number of individual piglets, all of which were given the same treatment except time of blood sampling. The first number in the piglet identification designates litter of origin (litter 1 or litter 2). Table 8 shows the study design. Figure S16. Relationship between terminal time (last serum sample before death) and serum Stx2 concentrations in neonatal gnotobiotic piglets after oral inoculation with EHEC O157:H7 strain EDL933 at 22–24 h of age (*n* = 29). Serum concentrations of two surviving piglets were plotted at 196–h post-inoculation. Table S1. Raw data file for the study. Table S2. Schedule for blood sampling to measure serum TMA-15 concentrations over time in neonatal gnotobiotic piglets after inoculation at 22–24 h of age with EHEC O157:H7 strain EDL933. Table S3. Serum concentrations of TMA-15 in individual piglets after single intraperitoneal administration of 3.0 mg/kg. Table S4. Schedule for blood sampling to measure Stx2 serum concentrations over time in neonatal gnotobiotic piglets after inoculation at 22–24 h of age with EHEC O157:H7 strain EDL933.

## Abbreviations

The following abbreviations are used in this manuscript:

A/E	Attaching-and-effacing
CNS	Central nervous system
CFU	Colony-forming units
EHEC	Enterohemorrhagic *Escherichia coli*
Gb_3_	Globotriaosylceramide
HUS	Hemolytic uremic syndrome
LS	Least squares
MAb	Monoclonal antibody
SAS	Statistical Analysis System
Stx	Shiga toxin
STEC	Shigatoxin-producing *Escherichia coli*
TMA	Thrombotic microangiopathy
TMA-15	Teijin monoclonal antibody-15 (Urtoxazumab)
