# Supplementary material for: Efficacy of Urtoxazumab (TMA-15 Humanized Monoclonal Antibody Specific for Shiga Toxin 2) Against Post-Diarrheal Neurological Sequelae Caused by Escherichia coli O157:H7 Infection in the Neonatal Gnotobiotic Piglet Model
Source: Toxins (Basel). 2017 Jan 26;9(2):49. doi: 10.3390/toxins9020049 (PMC5331429; doi:10.3390/toxins9020049)
Supplement: Supplementary file 1 [file toxins-09-00049-s001.pdf]

Supplementary Materials: Efficacy of Urtoxazumab (TMA-15 Humanized Monoclonal Antibody Specific for Shiga Toxin-2) Against Post-Diarrheal Neurological Sequelae Caused by *Escherichia coli* O157:H7 Infection in the Neonatal Gnotobiotic Piglet Model

Rodney A. Moxley, David H. Francis, Mizuho Tamura, David B. Marx, Kristina Santiago-Mateo and Mojun Zhao

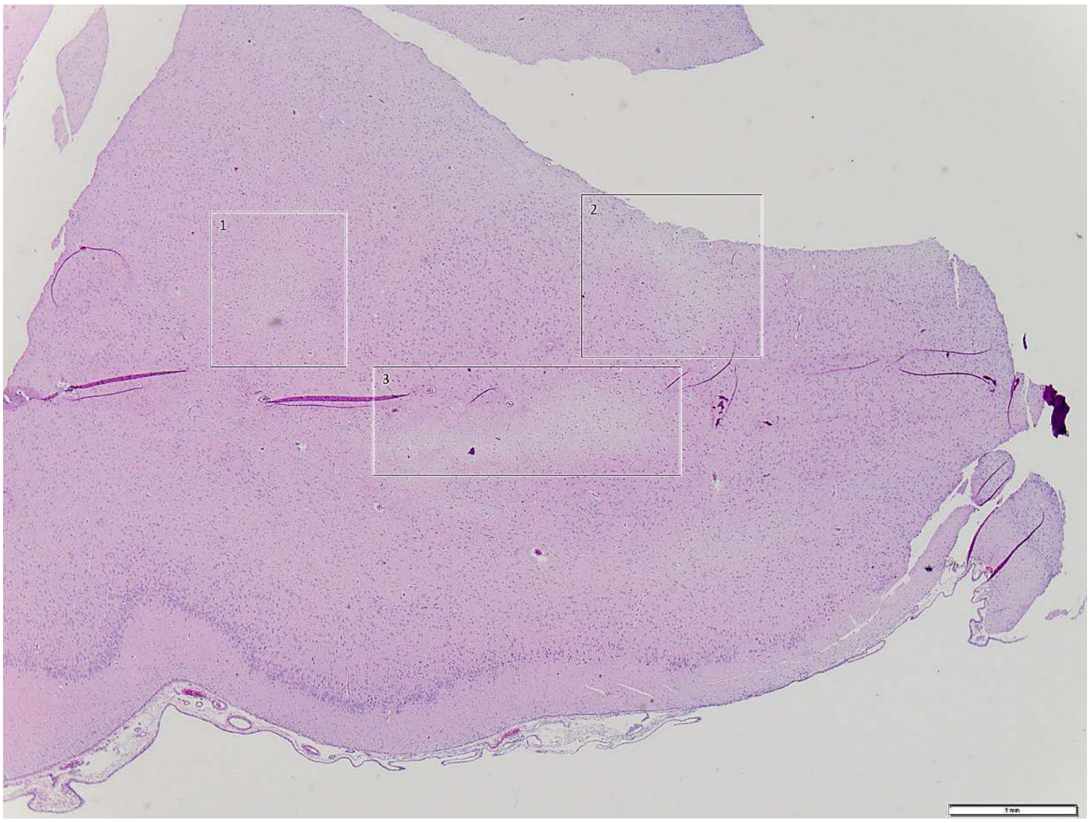

Figure S1. Low-magnification photomicrograph of cerebrum of placebo control piglet (No. 2F-1D) that was euthanatized when it became moribund 67 h post-inoculation with EHEC O157:H7 strain EDL933. Three foci of infarction (outlined by rectangles) are seen, which at this magnification appear as areas of pallor. Bar in lower right corner = 1 mm. Original objective magnification = 2×. Hematoxylin and eosin stain.

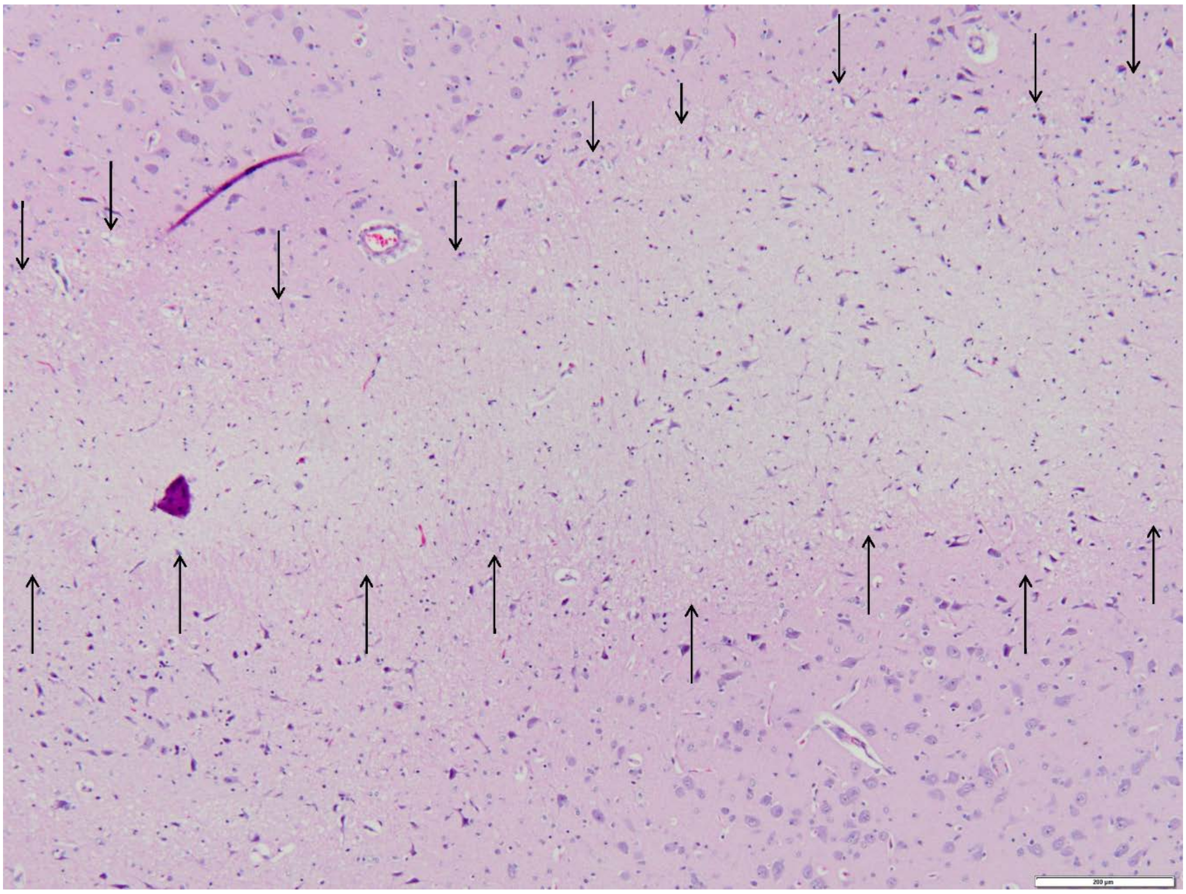

Figure S2. Higher magnification of area within box No. 3 in previous photomicrograph. Area of pallor includes vacuolated neuropil and shrunken, pyknotic cells. Borders of most severely affected area are denoted by arrows. Area in lower left corner below the arrows is also affected, perhaps with less severe neuropil vacuolation. Bar in lower right corner = 200 μm. Original objective magnification = 10×. Hematoxylin and eosin stain.

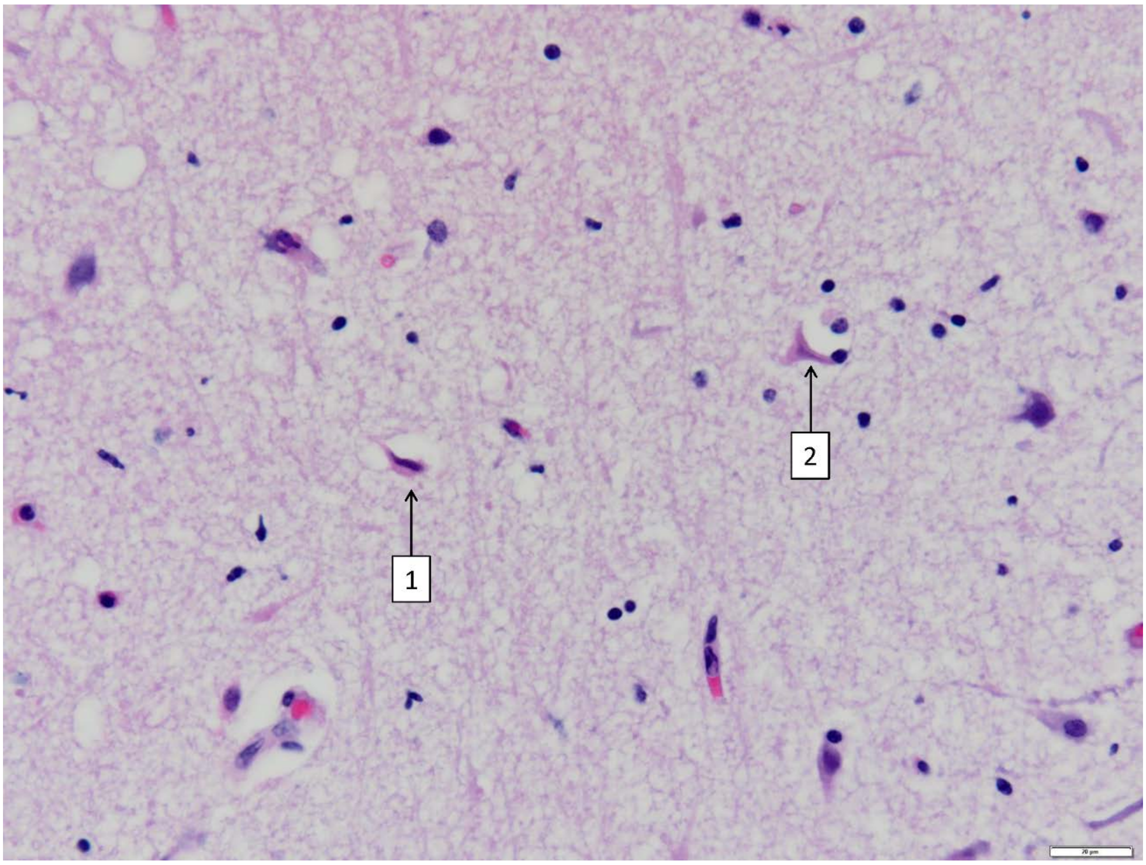

Figure S3. Higher magnification of area within infarct is shown in previous photomicrograph. Neuron No. 1 is necrotic, as evidenced by being shrunken with pyknotic nucleus and eosinophilic perikaryon. Neuron No. 2 is also necrotic, as evidenced by being shrunken with karyolytic nucleus and eosinophilic perikaryon. Other neurons in the figure (unlabeled) are necrotic. Neuropil is pale-staining and vacuolated. Bar in lower right corner = 20 μm. Original objective magnification = 60×. Hematoxylin and eosin stain.

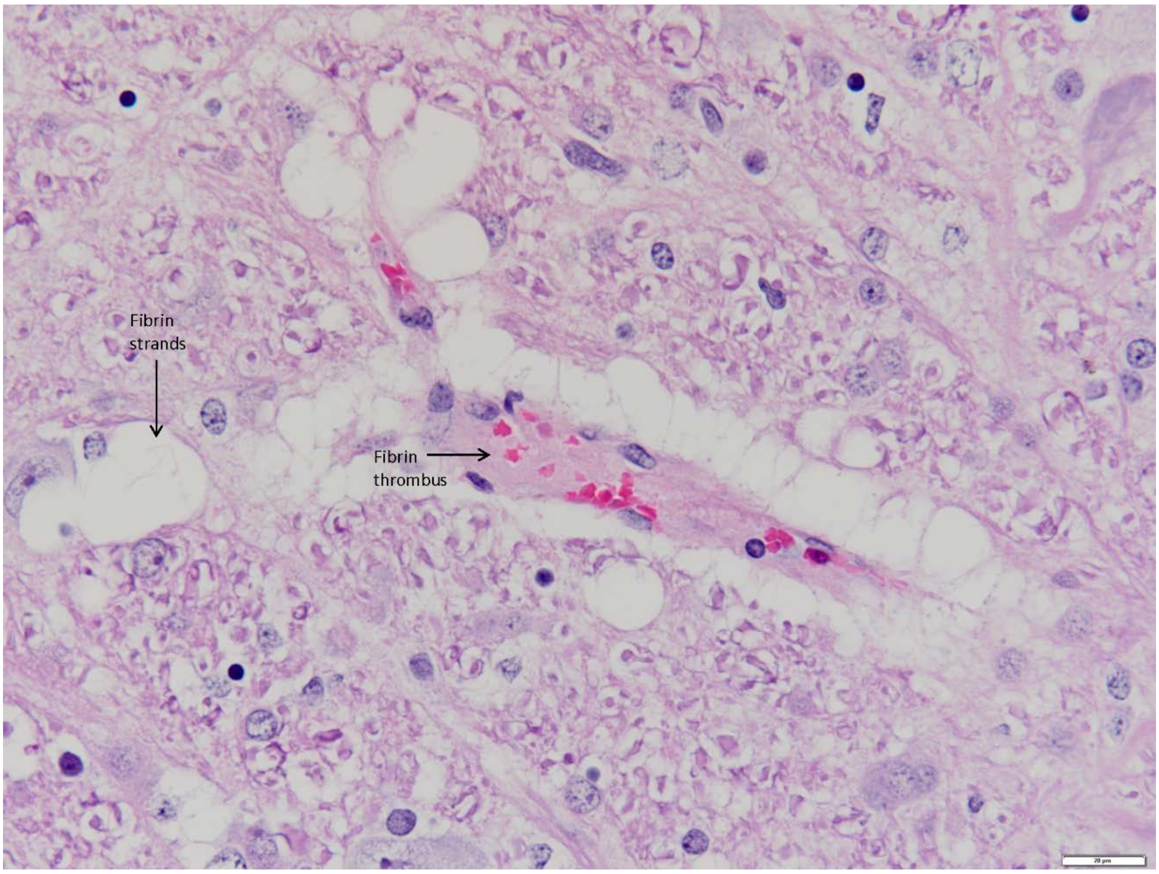

Figure S4. Photomicrograph of medulla oblongata of placebo control piglet (No. 2F1B) that was euthanatized when it became moribund 33 h after inoculation with EHEC O157:H7 strain EDL933. A fibrin thrombus (horizontal arrow with label) with entrapped red blood cells occluding a venule is seen in the center of the field. Fibrin strands are seen within a perivascular space (vertical arrow with label). Bar in lower right corner = 20 μm. Original objective magnification = 60×. Hematoxylin and eosin stain.

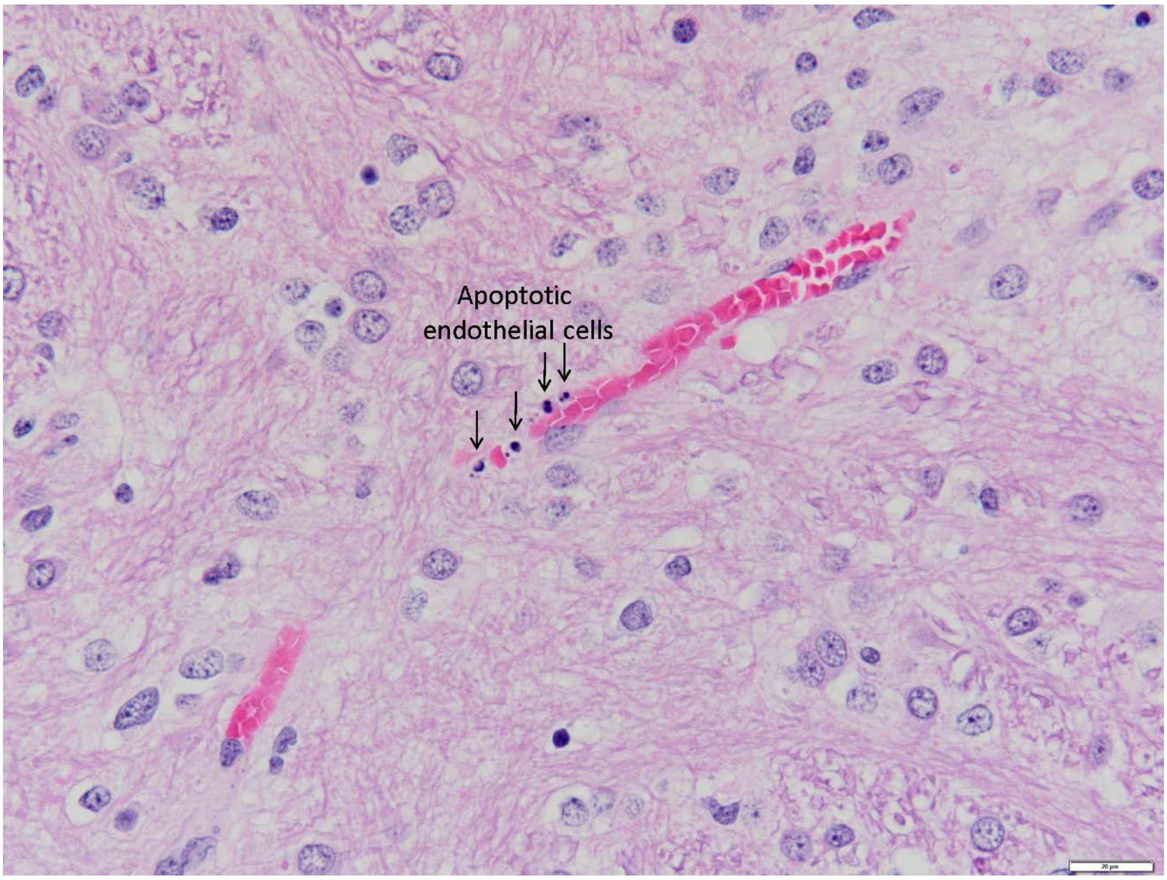

Figure S5. Photomicrograph of medulla oblongata is shown in previous figure. Endothelial cells within a venule show the morphologic hallmarks of apoptosis (arrows with label). Bar in lower right corner = 20 µm. Original objective magnification = 60×. Hematoxylin and eosin stain.

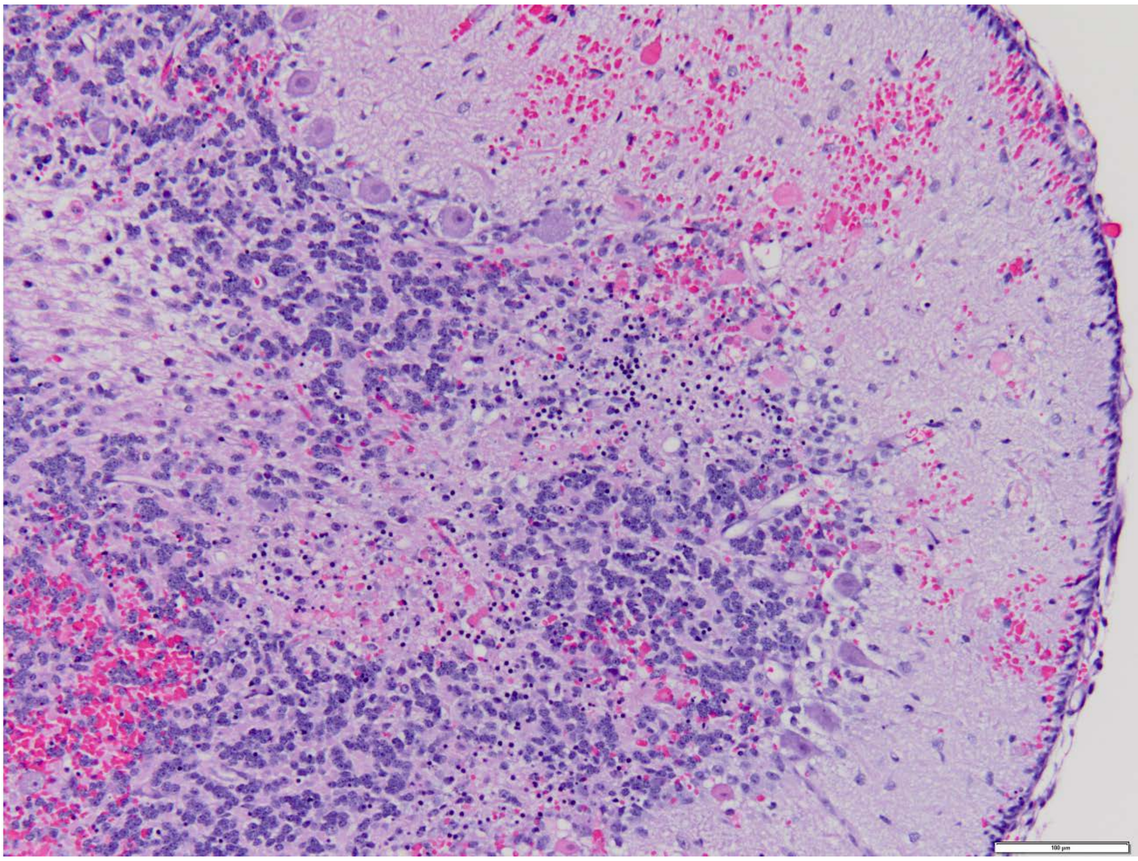

Figure S6. Photomicrograph of cerebellum of piglet (No. 3F1C) treated with TMA-15 (3.0 mg/kg) 24 h post-inoculation with EHEC O157:H7 strain EDL933. This piglet was the only one in the treatment group that became moribund (75 h post-inoculation). Lesions seen in this section include multiple coalescing infarcts (confluent necrosis) involving the molecular, granular, Purkinje and white matter layers, and associated hemorrhage. Bar in lower right corner = 100 μm. Original objective magnification = 20×. Hematoxylin and eosin stain.

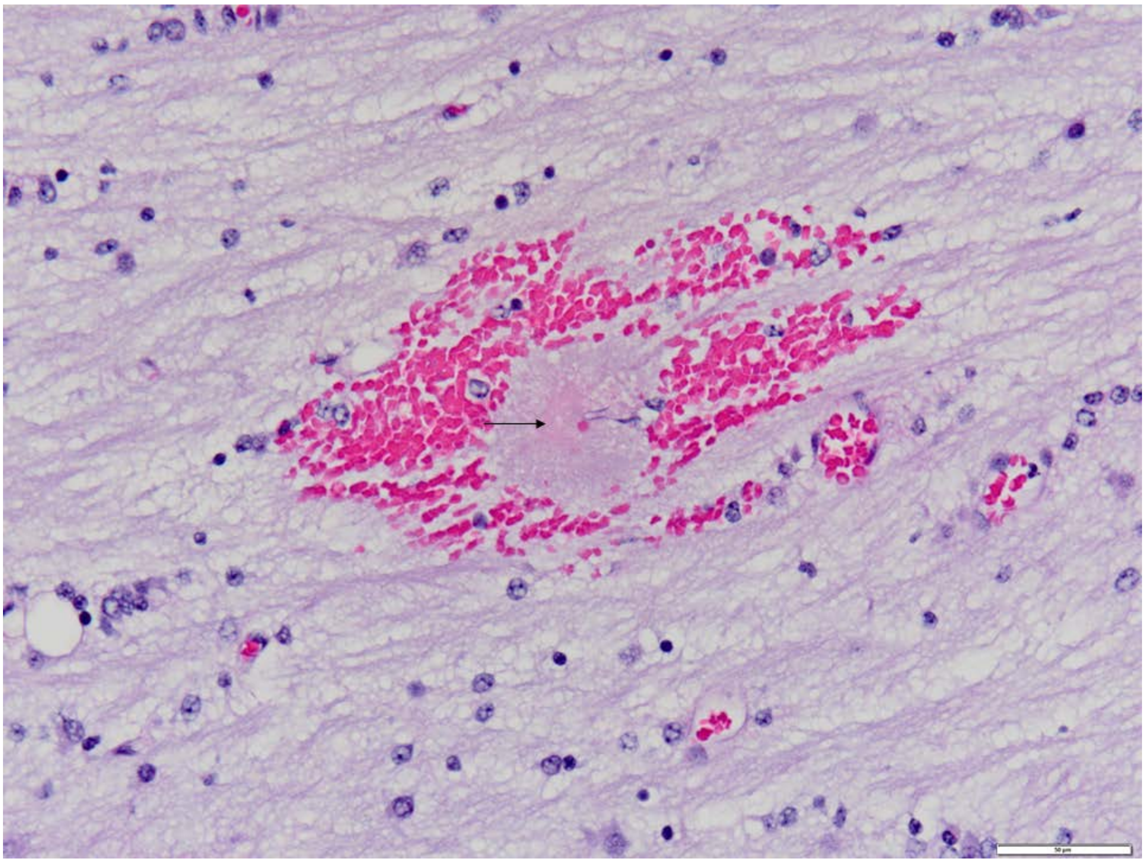

Figure S7. Photomicrograph of mesencephalon of placebo control piglet (No. 2F1B). A ring-shaped area of hemorrhage caused by circumferential bleeding around a necrotic arteriole is seen in the white matter in the center of the field. The material in the center of the lesion (arrow) is fibrin. Bar in lower right corner = 50 μm. Original objective magnification = 40×. Hematoxylin and eosin stain.

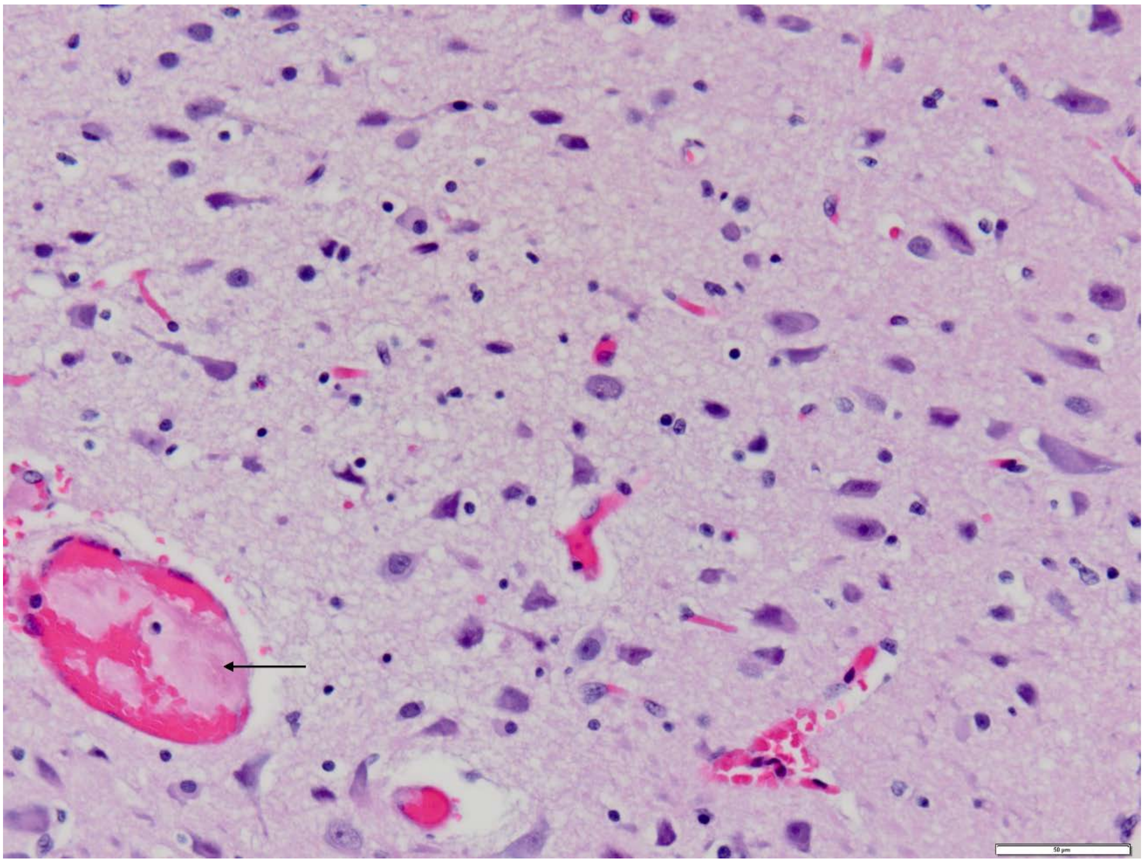

Figure S8. Photomicrograph of thalamus from same piglet is shown in previous figure. A thrombus (arrow) is seen in a venule in the lower left corner. The brain parenchyma immediately surrounding the thrombosed vessel is infarcted, as evidenced by necrotic neurons and other cells, and vacuolated neuropil. Bar in lower right corner = 50 μm. Original objective magnification = 40×. Hematoxylin and eosin stain.

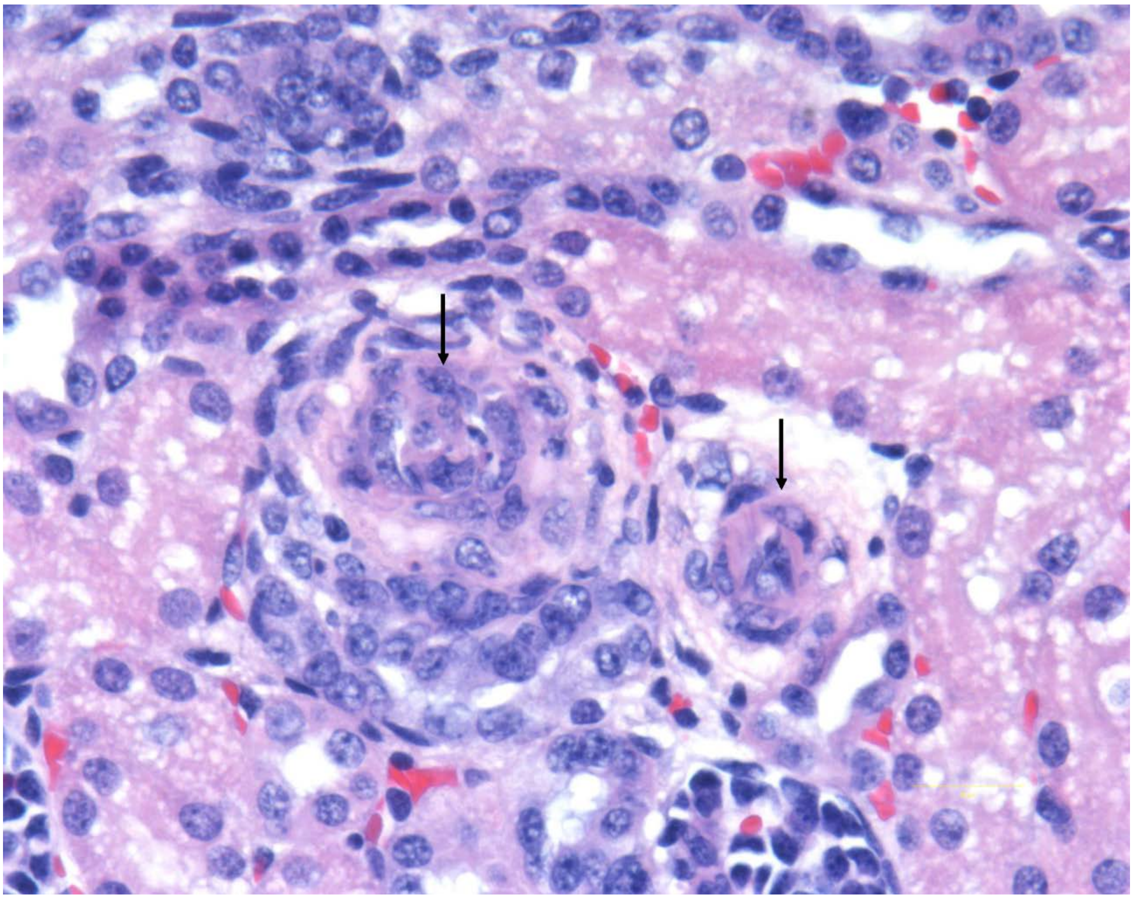

Figure S9. Photomicrograph of kidney of piglet 2F1C which was treated with TMA-15 at a dosage of 3 mg/kg BW and survived to 192 h post-inoculation with EHEC O157:H7 strain EDL933. Glomerulus in center of field contains capillary thrombi (horizontal arrows), and is hemorrhagic (vertical arrow). This piglet did not develop signs of CNS dysfunction and had no detectable brain lesions. Bar in lower right corner = 20 µm. Original objective magnification = 60×. Hematoxylin and eosin stain.

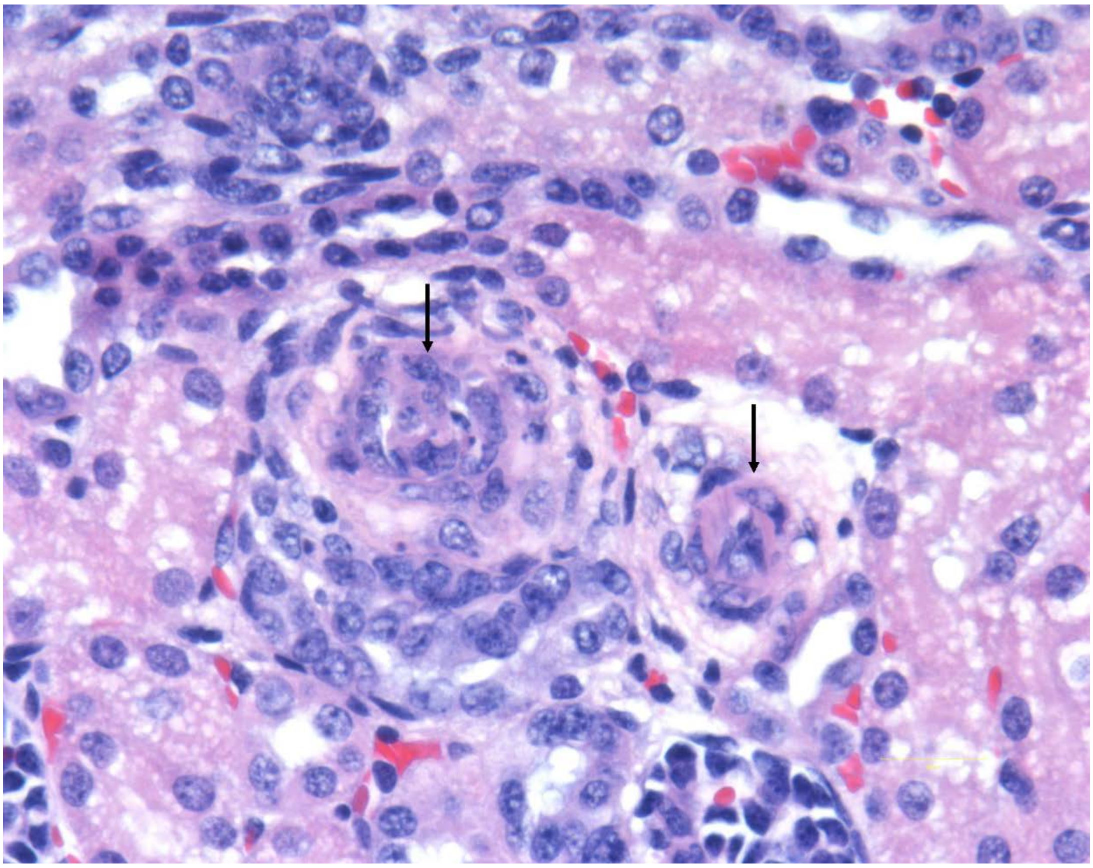

Figure S10. Photomicrograph of kidney of piglet 3F2D which was treated with TMA-15 at a dosage of 0.3 mg/kg BW and survived to 192 h post-inoculation with EHEC O157:H7 strain EDL933. Two arterioles in the center of the field (vertical arrows) have undergone proliferative microangiopathy, as evidenced by intimal proliferation that has thickened the walls and obliterated the lumens of these vessels. In addition, some myocytes in the tunica media have undergone fibrinoid change or coagulative necrosis. This piglet did not develop signs of CNS dysfunction and had no detectable brain lesions. Bar in lower right corner = 20 μm. Original objective magnification = 60×. Hematoxylin and eosin stain.

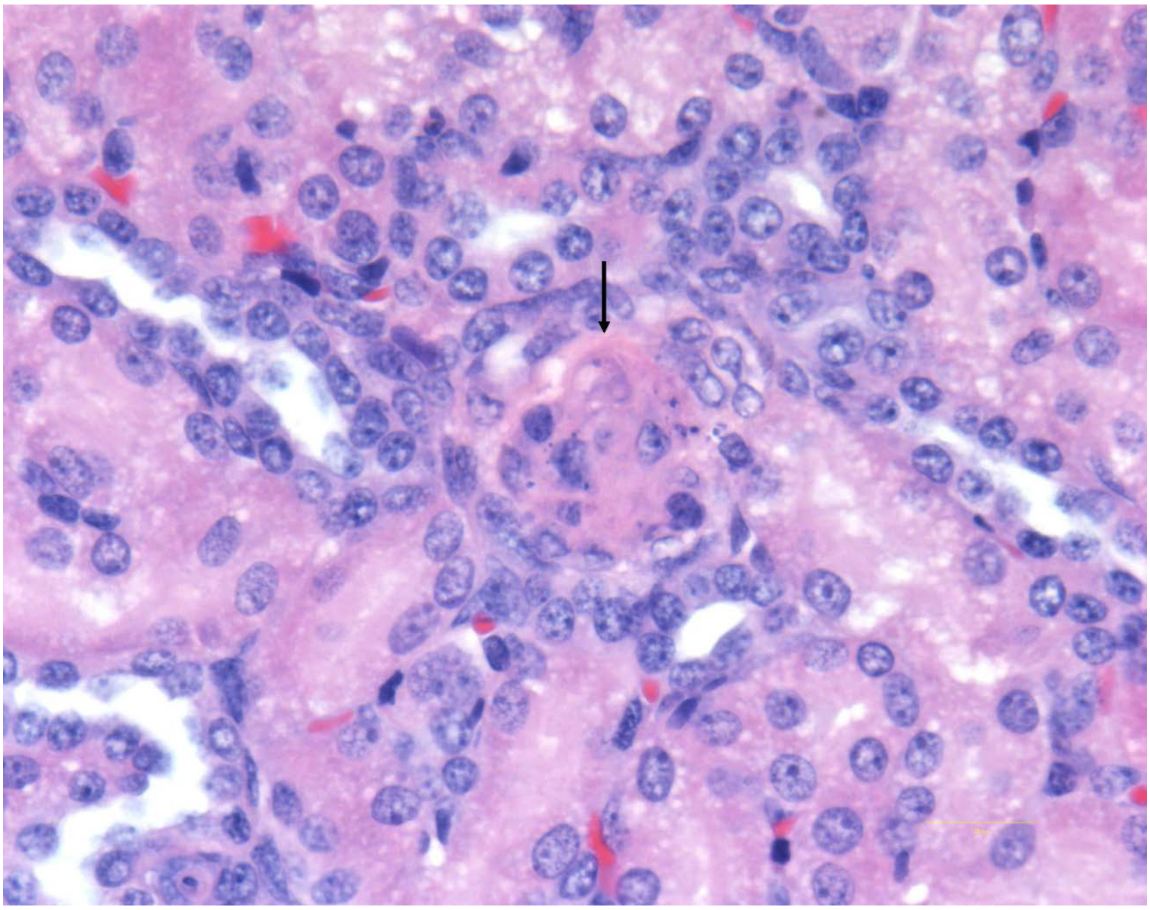

Figure S11. Photomicrograph of kidney of piglet 3F3D which was treated with TMA-15 at a dosage of 1.0 mg/kg BW and survived to 192 h post-inoculation with EHEC O157:H7 strain EDL933. An arteriole in the center of the field (vertical arrow) has undergone thrombotic microangiopathy with complete obliteration its lumen. Some myocytes in the tunica media have undergone fibrinoid change or coagulative necrosis with karyorrhexis and karyolysis of nuclei. This piglet did not develop signs of CNS dysfunction and had no detectable brain lesions. Bar in lower right corner = 20 μm. Original objective magnification = 60×. Hematoxylin and eosin stain.

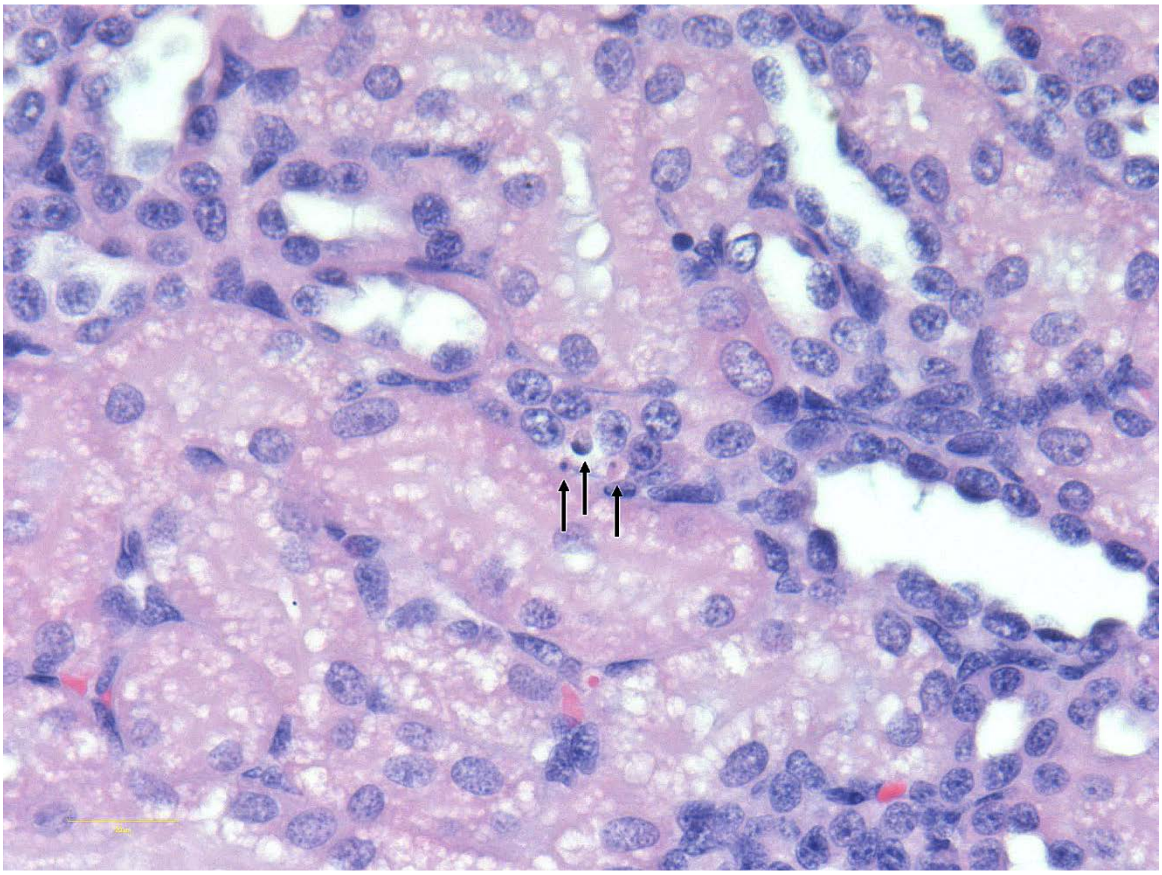

Figure S12. Photomicrograph of kidney of piglet 2F1B which was treated with placebo and died 67 h post-inoculation with EHEC O157:H7 strain EDL933. A renal tubule epithelial cell in the center of the field has undergone apoptosis, as evidenced by the presence of bleb fragments (arrows). Bar in lower left corner = 20 μm. Original objective magnification = 60×. Hematoxylin and eosin stain.

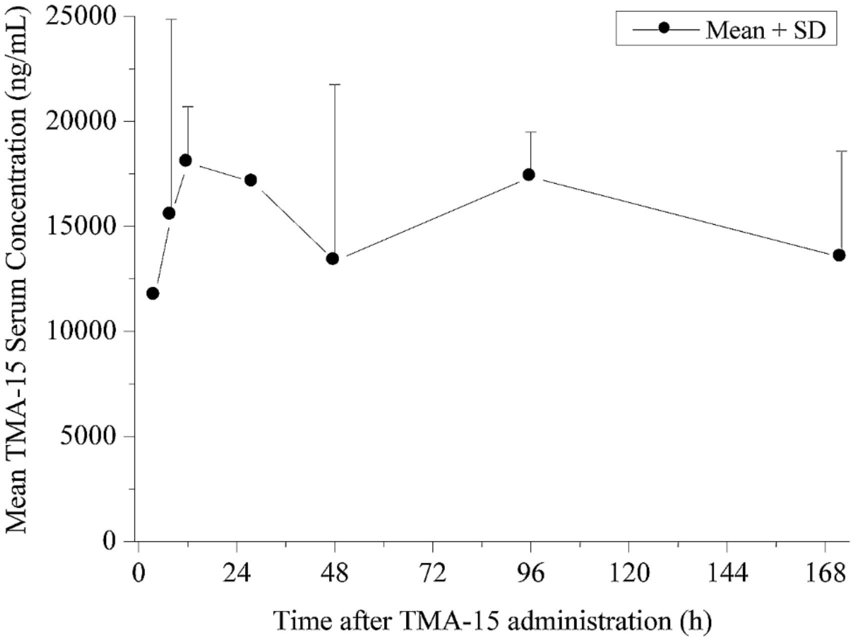

Figure S13. Mean serum TMA-15 concentrations (ng/mL) over time in neonatal gnotobiotic piglets inoculated with EHEC O157:H7 and treated by intraperitoneal injection with TMA-15 (3.0 mg/kg) 24 h after bacterial inoculation.

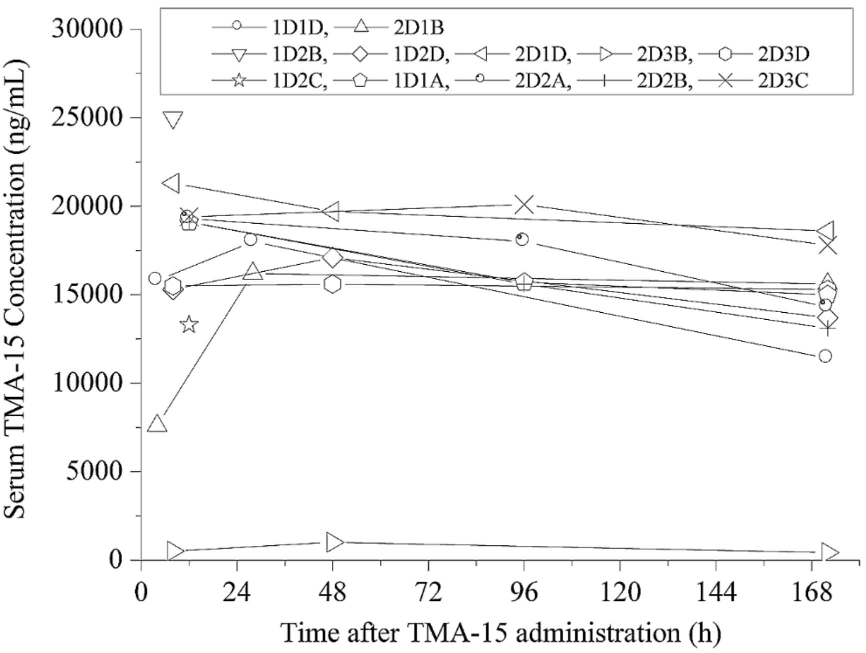

Figure S14. Serum TMA-15 concentrations (ng/mL) over time in individual neonatal gnotobiotic piglets inoculated with EHEC O157:H7 and treated by intraperitoneal injection with TMA-15 (3.0 mg/kg) 24 h after bacterial inoculation. Key to piglet ID is shown in Table S3.

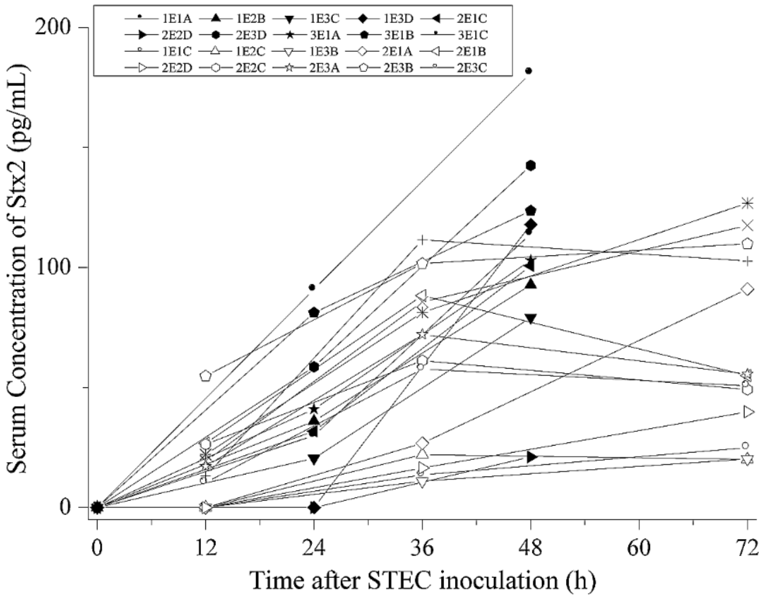

Figure S15. Serum Stx2 concentrations over time in individual neonatal gnotobiotic piglets after oral inoculation with EHEC O157:H7 strain EDL933 at 22–24 h of age. The key in the upper left hand corner shows the identification number of individual piglets, all of which were given the same treatment except time of blood sampling. The first number in the piglet identification designates litter of origin (litter 1 or litter 2). Table S4 shows the study design.

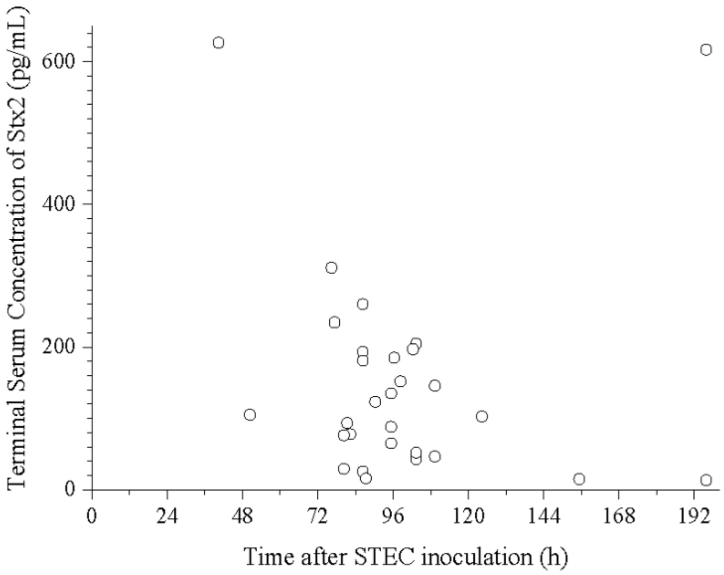

Figure S16. Relationship between terminal time (last serum sample before death) and serum Stx2 concentrations in neonatal gnotobiotic piglets after oral inoculation with EHEC O157:H7 strain EDL933 at 22–24 h of age (n = 29). Serum concentrations of two surviving piglets were plotted at 196–h post-inoculation.

Table S1. Raw data file for the study.

Pig#	Litter	Treatment	Hrs Survived	Survived to 192 h	CNS Signs	BW-TMA @trt	BW @ PM	%Wt Chg	%Wt Chg/h	Brain Lesion Scores—Area of Brain (#1–5) #1 (Med)	#2 (Cbel)	#3 (Mes)	#4 (Thal)	#5 (Cbru)	TOTAL	Death	Brain Lesions
1F1C	1	Placebo	71	0	1	1.24	1.20	−3.23	−0.05	1	1	4	4	4	14	1	1
1F2A	1	Placebo	82	0	1	1.12	1.08	−3.57	−0.04	2	7	7	7	7	30	1	1
2F1B	2	Placebo	33	0	1	1.10	1.10	0.00	0.00	2	7	4	4	4	21	1	1
2F1D	2	Placebo	67	0	1	1.74	1.80	3.45	0.05	1	4	4	7	0	16	1	1
3F1D	3	Placebo	66	0	1	1.52	1.60	5.26	0.08	5	4	1	11	4	25	1	1
3F2B	3	Placebo	75	0	1	0.86	0.82	−4.65	−0.06	4	4	10	11	11	40	1	1
4F1C	4	Placebo	137	0	1	1.48	1.66	12.16	0.09	11	10	10	10	1	42	1	1
4F3A	4	Placebo	149	0	1	0.98	1.04	6.12	0.04	0	11	1	0	0	12	1	1
5F1B	5	Placebo	192	1	1	1.24	1.64	32.26	0.17	10	10	10	10	0	40	0	1
5F2A	5	Placebo	112	0	1	1.22	1.34	9.84	0.09	10	4	10	10	1	35	1	1
				1/10			Mean	5.76	0.04	4.60	6.20	6.10	7.40	3.20	27.50	9/10	10/10
							SEM	3.46	0.02	1.33	1.05	1.19	1.18	1.14	3.63		
1F1B	1	0.3 mg/kg	127	0	1	1.38	1.38	0.00	0.00	1	10	4	11	4	30	1	1
1F2B	1	0.3 mg/kg	63	0	1	1.40	1.34	−4.29	−0.07	2	4	10	10	10	36	1	1
3F1B	3	0.3 mg/kg	192	1	0	1.28	1.76	37.50	0.20	2	NA	2	0	0	4	0	1
3F2D	3	0.3 mg/kg	192	1	0	1.26	1.72	36.51	0.19	3	4	3	10	1	21	0	1
3F3C	3	0.3 mg/kg	59	0	1	0.96	0.96	0.00	0.00	10	10	10	11	10	51	1	1
4F1A	4	0.3 mg/kg	192	1	0	1.60	2.02	26.25	0.14	0	0	0	0	0	0	0	0
4F2B	4	0.3 mg/kg	192	1	0	1.26	1.72	36.51	0.19	0	0	0	0	0	0	0	0
4F2D	4	0.3 mg/kg	192	1	0	1.04	1.54	48.08	0.25	0	0	0	0	0	0	0	0
4F3D	4	0.3 mg/kg	192	1	0	1.54	1.94	25.97	0.14	0	0	0	0	0	0	0	0
5F1A	5	0.3 mg/kg	192	1	0	1.50	2.00	33.33	0.17	0	0	0	0	0	0	0	0
5F2D	5	0.3 mg/kg	192	1	0	1.72	2.20	27.91	0.15	0	0	0	0	0	0	0	0
5F3A	5	0.3 mg/kg	192	1	0	1.54	2.00	29.87	0.16	0	0	0	0	0	0	0	0
				9/12			Mean	24.80	0.13	1.50	2.55	2.42	3.50	2.08	11.83	3/12	5/12
							SEM	4.90	0.03	0.83	1.21	1.10	1.49	1.12	5.20		
1F2C	1	1 mg/kg	50	0	1	1.02	1.04	1.96	0.04	1	1	1	10	10	23	1	1
3F1A	3	1 mg/kg	192	1	0	1.52	2.12	39.47	0.21	1	0	0	4	0	5	0	1
3F2C	3	1 mg/kg	192	1	1	1.30	1.94	49.23	0.26	0	0	0	0	0	0	0	0
3F3A	3	1 mg/kg	31	0	1	0.80	0.78	−2.50	−0.08	3	4	3	7	7	24	1	1
3F3D	3	1 mg/kg	192	1	0	1.40	2.06	47.14	0.25	0	0	0	0	0	0	0	0
4F1D	4	1 mg/kg	192	1	0	1.14	1.58	38.60	0.20	0	0	0	0	0	0	0	0
4F3B	4	1 mg/kg	192	1	0	1.30	1.80	38.46	0.20	0	0	0	0	0	0	0	0
4F2C	4	1 mg/kg	192	1	0	1.52	2.02	32.89	0.17	0	0	0	0	0	0	0	0
5F1C	5	1 mg/kg	192	1	0	1.72	2.24	30.23	0.16	0	0	0	0	0	0	0	0
5F2C	5	1 mg/kg	192	1	0	1.60	2.04	27.50	0.14	0	0	0	0	0	0	0	0
5F3B	5	1 mg/kg	192	1	0	1.42	1.86	30.99	0.16	0	0	0	0	0	0	0	0
				9/11			Mean	30.36	0.15	0.45	0.45	0.36	1.91	1.55	4.73	2/11	3/11
							SEM	5.01	0.03	0.28	0.37	0.28	1.07	1.06	2.84		
1F1A	1	3 mg/kg	64	0	1	1.14	1.08	−5.26	−0.08	2	2	2	10	4	20	1	1
1F1D	1	3 mg/kg	44	0	1	1.54	1.54	0.00	0.00	1	1	8	8	7	25	1	1
2F1A	2	3 mg/kg	192	1	0	1.66	2.14	28.92	0.15	1	1	0	1	0	3	0	1
2F1C	2	3 mg/kg	192	1	0	1.62	2.28	40.74	0.21	0	0	0	0	0	0	0	0
3F1C	3	3 mg/kg	75	0	1	1.24	1.24	0.00	0.00	4	10	10	11	10	45	1	1

3F2A	3	3 mg/kg	192	1	0	1.26	1.76	39.68	0.21	0	0	0	0	0	0	0	0
3F3B	3	3 mg/kg	59	0	1	1.14	1.15	0.88	0.01	0	4	11	11	11	37	1	1
4F1B	4	3 mg/kg	192	1	0	1.64	2.08	26.83	0.14	0	0	0	0	0	0	0	0
4F2A	4	3 mg/kg	192	1	0	1.44	1.88	30.56	0.16	0	0	0	0	0	0	0	0
4F3C	4	3 mg/kg	192	1	0	0.68	1.14	67.65	0.35	0	0	0	0	0	0	0	0
5F1D	5	3 mg/kg	192	1	0	1.14	1.66	45.61	0.24	0	0	0	0	0	0	0	0
5F2B	5	3 mg/kg	192	1	0	1.38	1.84	33.33	0.17	0	0	0	0	0	0	0	0
				8/12			Mean	25.74	0.13	0.67	1.50	2.58	3.42	2.67	10.83	4/12	5/12
							SEM	6.49	0.04	0.36	0.85	1.26	1.42	1.23	4.78		

Pig #: Identification number for piglet. Brain Lesion Score (#1-5) = Coronal section areas (reference points) as described in Reference 19: 1 = medulla oblongata (olivary nucleus); 2 = cerebellum (cerebellar peduncles); 3 = midbrain (corpora quadrigemina); 4 = cerebrum and thalamus (interthalamic adhesion); 5 = cerebrum (genu of corpus callosum); total = sum of scores of all five areas.

Table S2. Schedule for blood sampling to measure serum TMA-15 concentrations over time in neonatal gnotobiotic piglets inoculated with EHEC O157:H7 strain EDL933 *.

Group	Dosage Level of TMA-15 (mg/kg)	Timing of Blood Sampling	Gender	Survival after Inoculation †		
				Litter 1	Litter 2	Total
1	-	-		0/2	0/2	0/4
2	3	4 and 28 h post dosing and at euthanasia	Both	1/2	1/3	2/5
3	3	8 and 48 h post dosing and at euthanasia	Sexes	1/2	3/3	4/5
4	3	12 and 96 h post dosing and at euthanasia		0/1	4/4	4/5

* Piglets were orally inoculated at 22–24 h of age with 3 × 10^9 CFU EDL933, administered placebo or TMA-15 24 h after inoculation, and monitored for 192 h after inoculation. † Number of piglets surviving for 192 h/number inoculated.

Table S3. Serum concentrations of TMA-15 in individual piglets after single intraperitoneal administration of 3.0 mg/kg.

**Group 1**				
Animal No.	Bleeding Time (Hourss after STEC Inoculation)			
	59	89	103	169
1D1C	BLQ	-	-	-
1D2A	-	BLQ	-	-
2D3A	-	-	BLQ	-
2D2D	-	-	-	BLQ
**Group 2**				
Animal No.	Time after TMA-15 Dosing (h)			
	4	28	53**	59**
1D1A*	564	-		605
1D1D	15800	18000		-
2D1B	7600	16200		-
2D1C*	258	-	312	
2D2C*	118	BLQ		
Mean	11700	17100		n.c.
SD	n.c.	n.c.		n.c.
**Group 3**				
Animal No.	Time after TMA-15 Dosing (h)			
	8	46**	48	192
1D2B	25000	24600	-	-
1D2D	15300	-	17100	13700
2D1D	21300	-	19700	18600
2D3B	510	-	1010	422
2D3D	15500	-	15600	15300
Mean	13153	n.c.	13353	12006
SD	9335.8	n.c.	8400.9	7987.3
**Group 4**				
Animal No.	Time after TMA-15 Dosing (h)			
	12	53**	96	192
1D2C	13300	17700	-	-
2D1A	19100	-	15700	15000
2D2A	19300	-	18000	14300
2D2B	19100	-	15600	13100

2D3C	19400	-	20100	17800
Mean	18040	n.c.	17350	15050
SD	2652.9	n.c.	2142.4	1994.2

Piglets in Group 1 were not treated by TMA-15. Piglets in Group 2-4 were inoculated with EHEC O157:H7 strain EDL933 24 h prior to TMA-15 treatment (3 mg/kg, intraperitoneal injection). -: Sample was not taken. BLQ: less than 100 pg/mL, treated as 0. SD: standard deviations. n.c.: Not calculated.*: Anomalous serum concentration profiles, excepted from descriptive statistics. **: Terminal time

Table S4. Schedule for blood sampling to measure Stx2 serum concentrations over time in neonatal gnotobiotic piglets after inoculation at 22–24 h of age with EHEC O157:H7 strain EDL933.

Group	Timing of Blood Sampling	Gender	Litter #1	Litter #2	Litter #3	Total
1	At euthanasia	Both	$n = 2$	$n = 2$	$n = 2$	$n = 6$
2	Before and 24, and 48 h, after EHEC inoculation and at euthanasia	Both	$n = 4$	$n = 3$	$n = 3$	$n = 10$
3	12, 36, and 72 h, after EHEC inoculation and at euthanasia	Both	$n = 3$	$n = 7$	$n = 3$	$n = 13$
